# Model-Informed Optimization of a Pediatric Clinical Pharmacokinetic Trial of a New Spironolactone Liquid Formulation

**DOI:** 10.3390/pharmaceutics13060849

**Published:** 2021-06-08

**Authors:** Manasa Tatipalli, Vijay Kumar Siripuram, Tao Long, Diana Shuster, Galina Bernstein, Pierre Martineau, Kim A. Cook, Rodrigo Cristofoletti, Stephan Schmidt, Valvanera Vozmediano

**Affiliations:** 1 Center for Pharmacometrics and Systems Pharmacology, Department of Pharmaceutics, University of Florida, Gainesville, FL 32827, USA; t.manasa@ufl.edu (M.T.); vijay.siripuram@cop.ufl.edu (V.K.S.); taolong@cop.ufl.edu (T.L.); RCristofoletti@cop.ufl.edu (R.C.); SSchmidt@cop.ufl.edu (S.S.); 2 PRA Health Sciences, Raleigh, NC 27612, USA; shusterdiana@prahs.com (D.S.); martineaupierre@prahs.com (P.M.); 3 Camargo Pharmaceutical Services, LLC., Blue Ash, OH 45242, USA; gbernstein@camargopharma.com; 4 Kiel Laboratories, Inc., Flowery Branch, GA 30542, USA; kacook3@outlook.com

**Keywords:** spironolactone, pediatrics, model informed drug development, better medicines for children, pharmacometrics, pediatric drugs

## Abstract

Quantitative pharmacology brings important advantages in the design and conduct of pediatric clinical trials. Herein, we demonstrate the application of a model-based approach to select doses and pharmacokinetic sampling scenarios for the clinical evaluation of a novel oral suspension of spironolactone in pediatric patients with edema. A population pharmacokinetic model was developed and qualified for spironolactone and its metabolite, canrenone, using data from adults and bridged to pediatrics (2 to <17 years old) using allometric scaling. The model was then used via simulation to explore different dosing and sampling scenarios. Doses of 0.5 and 1.5 mg/kg led to target exposures (i.e., similar to 25 and 100 mg of the reference product in adults) in all the reference pediatric ages (i.e., 2, 6, 12 and 17 years). Additionally, two different sampling scenarios were delineated to accommodate patients into sparse sampling schemes informative to characterize drug pharmacokinetics while minimizing phlebotomy and burden to participating children.

## 1. Introduction

Spironolactone (SPIR) and two of its metabolites (7a-thiomethyl-spironolactone (TMS) and canrenone (CAN)) are aldosterone antagonists that bind to cytoplasmic mineralocorticoid receptors in the distal tubules of the kidney and promote sodium and water excretion as well as potassium retention. As such, these compounds are considered potassium sparing diuretics [1]. SPIR (Aldactone^®^ tablets) is approved by the US FDA for use in adults to treat several conditions, including severe heart failure (New York Health Association (NYHA) Class III/IV), edema due to cirrhosis or nephrotic syndrome, essential hypertension, and primary hyperaldosteronism [2]. SPIR has been administered off-label to pediatric patients since 1964 [3]. Dosing guidelines have been published by the World Health Organization [4] and the British National Formulary for Children [5] by age group and indication as SPIR is used in the pediatric population to treat edematous conditions such as congestive heart failure, ascites, and nephrotic syndrome [3]. As SPIR is not commercially available in a liquid oral dosage form, several liquid extemporaneous formulations of SPIR have been developed, including stability data, and are used in pediatric patients [6]. CaroSpir^®^ is an oral suspension of SPIR developed by CMP Pharma and offers the benefits of convenience, improved availability and stability, formulation consistency, and lack of compounding errors when compared to the extemporaneous formulations currently used. Currently, there are no FDA-approved pediatric indications for SPIR (administered as either Aldactone^®^ tablets or CaroSpir^®^ oral suspension).

Both the FDA and EMA approaches to pediatric development consider the possibility of extrapolation from adult or older children data if (1) there is a similar progression of the disease, (2) there is a similar response to treatment, and (3) there is a similar exposure or concentration response relationship [7]. In the specific case of SPIR, it is not possible to assume similar disease progression and response to intervention between pediatrics and adults, and therefore pharmacokinetic/pharmacodynamic (PK/PD) studies are needed to appropriately define dose recommendations in this population. The clinical development program of CaroSpir^®^ in adults relied on two comparative bioavailability (BA) studies and one food effect study to bridge the safety, PK, and efficacy from FDA-approved reference listed drug (RLD) Aldactone^®^ tablets (NDA 12151 approved in 1960) to the oral suspension. The suspension of SPIR demonstrated a higher relative bioavailability compared to Aldactone^®^ tablets, and the magnitude of difference in relative BA increased with higher doses. When a 25 mg dose was administered, the oral suspension showed 21% and 15% higher Cmax and AUC0-∞, respectively, compared to the same dose of RLD. Further, when 100 mg was administered, the oral suspension showed 65% and 37% higher Cmax and AUC0-∞, respectively, compared to the 100 mg dose of the RLD. Based on these results, it was likely that doses of the oral suspension greater than 100 mg could result in SPIR concentrations higher than expected relative to Aldactone^®^ tablets. Therefore, CaroSpir^®^ was approved in adults for those indications that required doses of 100 mg or lower, including edema caused by liver cirrhosis, heart failure, and hypertension.

SPIR is poorly soluble in water, and the administration of the drug with food increases the BA, probably as a consequence of improved absorption, but also a possible decrease in the first-pass hepatic metabolism [8,9,10,11]. This is in line with the findings in the food effect study [12], where a high fat and high calorie meal (57% of the ~1000 kcal of the meal from fat) increased the BA of SPIR (as measured by AUC0-∞) by approximately 90% [12,13,14,15]. SPIR is rapidly metabolized hepatically into a number of metabolites, although the main enzymes involved in its complex metabolism are still understudied. At therapeutic concentrations, both SPIR and CAN are highly bound to plasma proteins [16]. In patients with cirrhosis, SPIR and CAN’s plasma half-life (t1/2) increase from 1.4 and 16.5 h to 9 and 58 h, respectively [17].

The impact of renal impairment, age, sex, or race on SPIR PK have not been specifically studied in adults, nor has the safety, efficacy, and PK been well characterized in the pediatric population (despite its off-label use in these patients for a number of years). Due to the lack of safety, efficacy, and PK data in pediatric patients, as well as gaps in the characterization of SPIR PK in adult patients, there are no specific dosing recommendations provided in the Aldactone^®^ tablet prescribing information for pediatric subjects. Considering the potential benefits of an oral suspension of SPIR in pediatrics and the absence of pediatric prescribing information on the RLD label, a pediatric drug development program for CaroSpir^®^ was proposed to evaluate the safety, effectiveness, and PK of the new formulation for the treatment of pediatric patients aged 0 to <17 years old with edematous conditions associated with heart failure and liver cirrhosis. A model-based optimization was then proposed in order to support the dose selection and PK sampling scheme for a pediatric PK/PD clinical study in the two target indications. The specific aims of the present study were: (1) to characterize SPIR and its metabolite CAN PK in healthy adults through population modeling of the data from two comparative bioavailability phase I studies and one food effect study, (2) to bridge the PK model to pediatrics (2 to <17 years old) using allometric scaling to support dose selection recommendations in this population, and (3) to optimize the PK sampling scheme for SPIR and CAN. Due to the important gaps of knowledge on SPIR PK, a simulation-based evaluation of possible what-if scenarios for non-cirrhotic and cirrhotic pediatric subjects receiving SPIR in fasted and fed states was performed using the pediatric model and the incorporation of PK changes under these clinical circumstances. In the future, when pediatric PK data is available for patients aged 2 to <17 years old, the proposed pediatric PK model described in this manuscript will be further refined to support dose selection and optimal PK sampling in pediatric patients aged 0 to <2 years old and inform a larger multiple dose PK/PD study in pediatric patients aged 0 to <17 years old.

## 2. Material and Methods

### 2.1. Study Design and General Overview of the Data Analysis Steps

SPIR and CAN plasma concentration data from two separate single-dose comparative bioavailability studies (063-15 and 064-15) and one food effect study (084-15) with CaroSpir^®^ oral suspension were used for model development and qualification in adults. The details of the study designs are summarized in Table 1.

All of the available data on SPIR oral suspension under fasted conditions were analyzed simultaneously. The studies were approved by the corresponding institutional review board (IRB) and conducted in accordance with the principles of the Declaration of Helsinki. All participants gave signed informed consent.

### 2.2. Population PK Analysis

The population analysis was aimed to develop a population PK (popPK) model able to adequately describe SPIR and CAN PK behavior in healthy adult subjects receiving the oral suspension and to use this model to extrapolate the PK to the pediatric population (2 to <17 years). The model building was performed in a step-wise fashion as follows: (1) proprietary data set placed into NONMEM data stream format, (2) building the pharmacostatistical model, (3) qualification of the model, and (4) extrapolation to the pediatric population using allometric scaling.

PK population modeling and simulations were performed using the NONMEM^®^ program (double precision, version 7.3 ICON Development Solutions, Ellicott City, Maryland) with the NMTRAN pre-processor and the ADVAN routines, mostly ADVAN13 with TOL = 6 for the parent metabolite model and $DES (differential equation) as required. Other supportive software used for data management, graphics, metadata handling, and plotting were R^®^ (version 3.6.0 [www.r-project.org (accessed on 27 February 2021)] running under Rstudio interface), Pirana v2.9.9 (http://www.pirana-software.com/ (accessed on 27 February 2021)) and Perl-Speaks-NONMEM v4.9.0 (https://uupharmacometrics.github.io/PsN (accessed on 27 February 2021)). Additional simulations for dose selection and what-if scenarios were performed using MATLAB R2020b (Mathworks^®^).

Model development and qualification (goodness of fit, visual predictive check (VPC) and bootstrapping) was done following standard and well-accepted population procedures and guidelines [18]. A detailed description of the population analysis is presented in Section S1 of the Supplementary Materials.

### 2.3. Extrapolation of the Parent Drug-Metabolite Model to Pediatrics

An allometric scaling approach was applied to bridge the parameters of the adult popPK model for SPIR and CAN to pediatric subjects of different ages, as shown in the following equation:

(1) $$P_{ped}{=P}_{adult}{\left(\frac{WT}{70}\right)}^{b}$$

where *P_ped_* is the parameter in pediatrics of different ages, *P_adult_* is the typical parameter value in adults from the population PK model described above, *WT* is the 50 percentile values of body weight taken from the CDC growth charts for pediatric subjects aged 2 years and older in the U.S. (both males and females), and *b* is the allometric exponent that will be 0.75 for both elimination and intercompartmental clearance and 1 for volumes of distribution [19,20,21]. Allometric scaling was done and implemented in MATLAB for simulation. Both, the interindividual and residual variability estimated in adults were carried out to pediatrics [22].

The popPK model for pediatrics was applied to explore the drug exposure using various dose strengths of SPIR oral suspension in pediatric subjects of different ages (both males and females) The model used for simulation in the pediatric population not only accounted for the mean behavior but also for random effects on the PK parameters, allowing the calculation of confidence intervals and thus the inference of PK outcomes within a statistical framework of 95% CI. Despite the available pediatric guidelines and adult clinical studies, target efficacious and safe exposures have not been well characterized in any of the populations, including healthy adult subjects as well as adult patients. As a consequence, the target exposure used initially for simulations was that observed in healthy adults in the clinical studies (Table 1). Predicted exposures in adult patients with liver cirrhosis based on PK changes reported in the literature were also used in the evaluation [17,23].

### 2.4. Dose Selection

The parent metabolite model built for the pediatric population, as described above, was used to simulate SPIR and CAN plasma profiles in pediatric patients of different ages receiving different doses of the oral suspension within the range 0.25–2 mg/kg. Moreover, the following was also considered for the pediatric dose selection: (1) existing guidelines and recommendations for SPIR administration in pediatric subjects with edematous conditions [4,5]; (2) differences in SPIR exposures when administered as an oral suspension with and without food, and differences in exposures when administered as tablets (Aldactone^®^) versus oral suspension (CaroSpir^®^); and (3) in vitro characterization of SPIR metabolism (i.e., main CYP enzymes identified as contributors to SPIR metabolism in in vitro studies, namely cytochrome CYP2C8 and CYP3A4/5, as well as Flavin-containing monooxygenases (FMOs), are already mature by the age of 2 years [24,25,26]).

Under the above considerations, a model-based dose selection strategy was followed using the allometric pediatric PK model, where different dosing scenarios with N = 200 subject per pediatric age (100 males and 100 females; 17, 12, 6, and 2 years old were selected as reference ages for dose evaluation) were simulated after the administration of SPIR oral suspension as single dose. The variability in the PK parameters quantified in adults was carried over to the pediatrics for the evaluation. The aim of the simulations was to find SPIR doses needed to achieve systemic exposures of SPIR and CAN comparable to those observed at 25 mg and 100 mg in healthy adults after a single dose, assuming these exposures are efficacious and safe in both adult and pediatric patients.

Additionally, the model was used to simulate SPIR and CAN exposures in pediatric subjects under different clinical scenarios such as the effect of cirrhosis and high-fat, high-calorie food by accounting for PK changes in these situations. The simulations were performed under the same design simulation settings as those used for the dose selection. The predicted exposures were compared with that in adults after 25 mg and 100 mg doses in the following conditions: (1) observed exposures in healthy adults in the fed state and (2) predicted exposures in adults with liver cirrhosis in both fasted and fed states. The purpose of this evaluation was to ensure that the selected doses for the pediatric clinical trial yield predicted exposures within the expected values in adults and minimized any possible safety concerns on the selected doses for the clinical trial.

### 2.5. Sampling Scheme Selection

The sampling scheme was developed based on the simulated pediatric PK profiles of subjects 2 to <17 years old, maximizing the information that could be obtained using the fewest number of blood samples, thus minimizing the burden on participating pediatric subjects. The PK sampling scheme was developed based on the following study design assumptions: a single- (period I) and multiple-dose (period II) clinical pediatric PK study in three pre-defined age groups (Group 1: 12 to <17 years, Group 2: 6 to <12 years, and Group 3: 2 to <6 years), where pediatric patients with edema due to cirrhosis or heart failure will not remain hospitalized during the study period. The aim of the work presented here was to select the doses for period I (i.e., single dose) based only on the PK component of the study.

Monte Carlo simulations were performed in NONMEM^®^ using the pediatric parent drug-metabolite model in virtual populations of 1000 pediatric subjects between 2 and <17 years old receiving the selected low dose (0.5 mg/kg) to evaluate the PK profile at both extremes of the age range. The simulated profiles were used to establish a range of times around maximum plasma concentrations (Tmax values) to guide the sampling schedule around the expected peak. The selection of the sampling times took into consideration that to completely describe a pharmacokinetic profile one must obtain a sufficient number of blood samples in the absorption, distribution, and elimination phases.

Samples collected during the first 6–8 h were aimed to characterize the absorption and formation of SPIR and CAN, respectively, as well as early distribution phases, where more variability was expected. The additional samples proposed were aimed to characterize the late distribution and elimination phases. Sampling windows were then created around the selected sampling intervals to facilitate the logistics of the pediatric trial, where samples can be taken at random. The model was used to reproduce the study design via simulation, and the output was then used to estimate the PK parameters using the model. The estimated parameters from the sparse sampling design were compared to those from the original model used for simulation. Additional information of the evaluation of the proposed study design to appropriately estimate the PK parameters in pediatrics is provided in Section S3 of the Supplementary Materials.

## 3. Results

### 3.1. Parent—Metabolite popPK Model in Adults

The final model of SPIR and CAN was developed based on the PK observations from N = 92 individuals receiving a single dose of 25 or 100 mg of SPIR oral suspension. All subjects were healthy adult male subjects of South Asian descent (Indian) of comparable characteristics, as summarized in Table 2.

**Figure 1:**
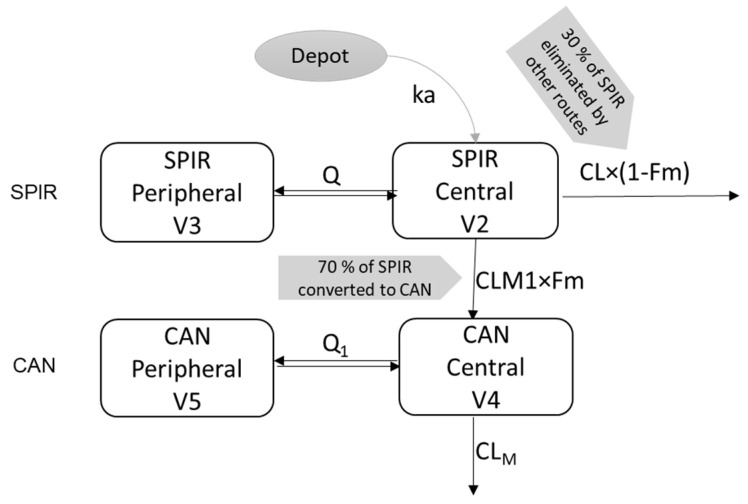
Structural PK model for the analysis of SPIR and CAN. Abbreviations: SPIR, spironolactone; CAN, canrenone; CLM1, SPIR clearance to CAN formation; CL, SPIR apparent clearance; V2, SPIR central compartment volume of distribution; Q, SPIR intercompartmental clearance; V3, SPIR peripheral compartment volume of distribution; Ka, SPIR absorption rate constant; CLM, CAN apparent clearance; V4, CAN central compartment volume of distribution; Q1, CAN intercompartmental clearance; V5, CAN peripheral compartment volume of distribution; Fm, fraction metabolized to CAN, fixed to 0.7; Spironolactone total clearance = CL × (1 − Fm) + CLM1 × Fm.

Based on both the visual inspection of SPIR and CAN PK data, as well as standard model evaluation criteria, a two-compartment model parameterized in terms of volumes of distribution and intercompartmental clearances was applied for the analysis of both analytes. The final model structure is shown in Figure 1.

This is a structurally locally identifiable model [27] parameterized in terms of SPIR clearance (CL), SPIR apparent clearance to CAN formation (CLM1), central volume of distribution (V2), intercompartmental clearance (Q), peripheral volume of distribution (V3), and absorption rate constant (ka) and lag time to explain a slight delay in the absorption. The metabolite was parameterized as CAN apparent clearance (CLM), central volume of distribution (V4), intercompartmental clearance (Q1), peripheral volume of distribution (V5), and fraction metabolized (Fm). It is to be noted that due to unavailability of intravenous PK data, bioavailability (F) of SPIR in the model was structurally unidentifiable; hence, a structurally locally identifiable model where F was indirectly estimated in the form of apparent parameters weighted/conditioned by F (for instance, CL/F for clearance of SPIR); for convenience of the notation, parameters for clearance and volume of distribution are mentioned without F; in the entire manuscript they represent apparent values of the respective parameters. Since the inclusion of delay in the absorption of SPIR led to improvement of objective function value (OFV), a lag time was included in the model. An exponential error model was used for interindividual variability of PK parameters. A proportional error model was selected for the residual variability based on the inspections of the model standard diagnostic plots. Subject number 8 from study 063-15 was excluded from the analysis due to protocol deviation (15 min deviation occurred in blood sample collection at 0.25 h due to cannula blockage in period II and the sample was collected at 0.5 h instead. As there was also a scheduled blood sample collection at 0.5 h, a total of two blood samples were collected one after the other at 0.5 h. When the samples were sent for bioanalysis, though these two samples were collected at the same time, i.e., at 0.5 h, because the samples were collected one after the other, they resulted in two different values for concentration at 0.5 h. Of the 24 subjects enrolled and randomized in study 084-15, only 23 subjects completed the clinical phase and were included in the analysis.

The popPK model parameter estimates for SPIR and CAN with the final model are listed in Table 3. The precision of parameters estimated was generally acceptable with a relative standard error (RSE) less than 30% for all structural and random parameters, except for the BSV on V4 and Q1 for which the RSE was less than 50%, suggesting acceptable precision for the estimated parameters using the first-order conditional estimation method with the interaction (FOCEI) method. Though the variability of the absorption rate constant was very large, the precision of the parameter estimate was acceptable. The interindividual variability was modeled for Ka, CL, V2, Q, V4, CLM, Q1, V5, and CLM1 with covariance between CL and V2. The distributions were generally normal for the interindividual variability of Ka, CL, V2, Q, V4, CLM, Q1, V5, and CLM1 (results not shown).

The goodness of fit plots (Figures S1–S6 in the Supplementary Materials) suggested acceptable fits of the data and no trend in the residuals. Moreover, the individual fits were acceptable, and the predicted concentration-time profiles represented well the observed concentrations in the individual plots (Figures S7 and S8 in the Supplementary Materials). The visual screening of covariates, including age, weight, body mass index, body surface, serum urea, serum creatinine, serum sodium, serum potassium, total proteins, and albumin, showed no apparent trend for the relationship between the individual residuals of the main SPIR and CAN PK parameters calculated by the base parent drug-metabolite model, which could be related to the homogeneity of the subjects’ demographic and clinical characteristics in studies 063-15, 064-15, and 084-15 (Figure S9 in the Supplementary Materials).

The final model qualification using VPC is shown in Figure S10 in the Supplementary Materials for the 25 and 100 mg of the oral suspension and for SPIR and CAN. Moreover, the results of the qualification of the final model using bootstrapping are shown in Table 3 (along with the PK parameters of the final model). The successful estimation accounted for 54% of 1000 bootstrap replicates, allowing for the estimation of the 0.5 and 99.5 percentiles (99% CI). The results of 540 successful runs were thus used for summary statistics. The relative error of bootstrap mean to original mean of each parameter was acceptable. The centers of distribution are generally comparable to the original mean values of final model. Above results suggest that the accuracy and robustness of the parameters estimated with the final parent drug-metabolite model are acceptable. In general, diagnostic plots did not reveal any model misspecification and estimated parameters were physiologically plausible. The model captured the central tendency of the data well and was deemed suitable to generate simulations.

### 3.2. Extrapolation of the Parent Metabolite Model to Pediatrics

The mean predicted PK parameters for SPIR and CAN in the pediatric population extrapolated from adults using allometric scaling and incorporated in the parent drug-metabolite model are depicted in Table 4.

### 3.3. Application of the Model to Select the Dose and Design the Pediatric Clinical Trial

The present section summarizes the results of the application of the model to (1) select a preliminary low and high dose for a single- and multiple-dose pediatric PK/PD clinical trial in subjects aged 2 to <17 years old and (2) propose a PK sampling schedule to adequately describe SPIR and CAN PK in this pediatric age range. As pediatric PK data becomes available from older age groups, the final parent drug-metabolite model will be refined to support dose selection in pediatric subjects aged 0 to <2 years old.

### 3.4. Dose Selection

**Figure 2:**
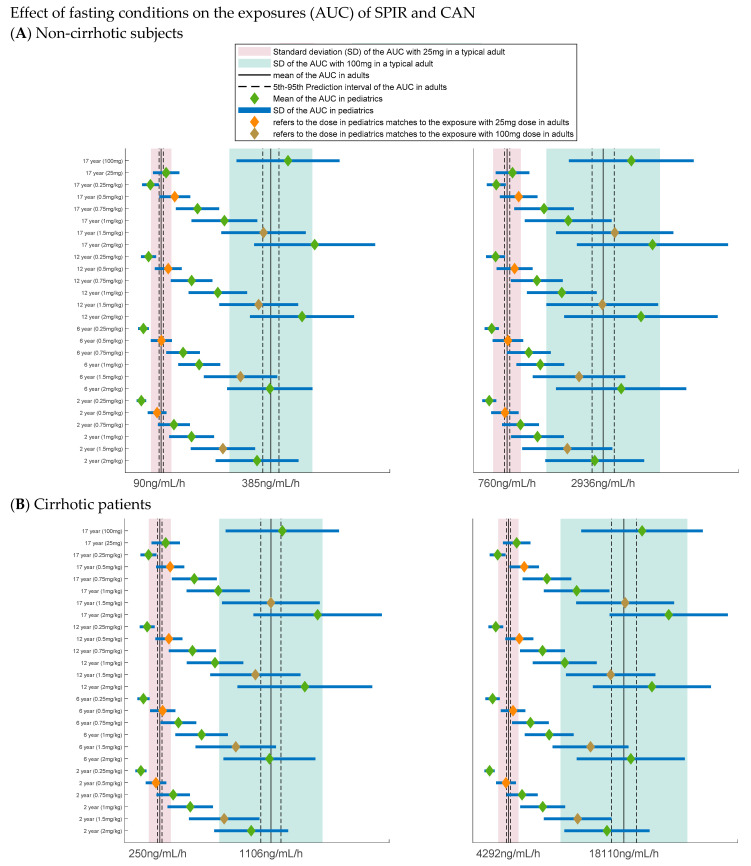
Results for what-if scenarios in fasted state. Forest plots showing the simulated exposures of SPIR and CAN in fasted state in subjects 2 to <17 years old administered the oral suspension as a single dose of 0.25, 0.5, 1, 1.5, and 2 mg/kg compared to that in typical adults administered 25 mg and 100 mg. (**A**) Non-cirrhotic pediatric subjects and (**B**) cirrhotic pediatric subjects. The green diamond and solid blue lines reflect the mean and (±) SD of AUCs in corresponding age groups; the black solid lines, two dashed lines, and bands indicate the mean, 5th, and 95th prediction interval and (±) SD of AUC (pink for 25 mg and green for 100 mg) in the reference adult, respectively. Reference adult: (**A**) fasted healthy adults and (**B**) fasted cirrhotic adult patients.

**Figure 3:**
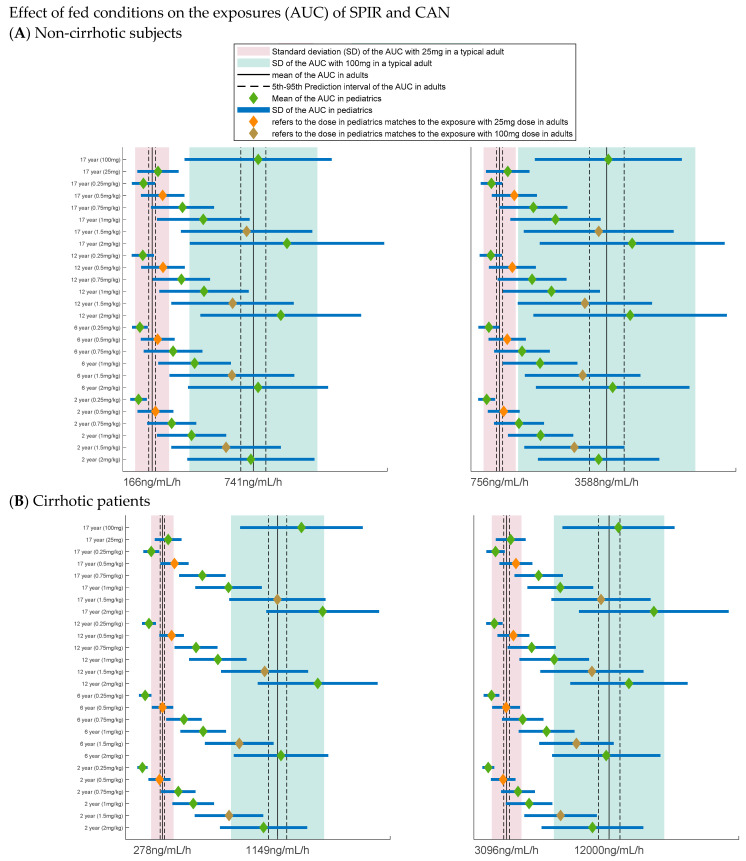
Results for what-if scenarios in fed state. Forest plots showing the simulated exposures of SPIR and CAN in fed state in subjects 2 to <17 years old administered the oral suspension as a single dose of 0.25, 0.5, 1, 1.5, and 2 mg/kg compared to that in typical adults administered 25 mg and 100 mg. (**A**) Non-cirrhotic pediatric subjects and (**B**) cirrhotic pediatric subjects. The green diamond and solid blue lines reflect the mean and (±) SD of AUCs in corresponding age groups; the black solid lines, two dashed lines, and bands indicate the mean, 5th, and 95th prediction interval and (±) SD of AUC (pink for 25 mg and green for 100 mg) in the reference adult, respectively. Reference adult: (**A**) fed healthy adults and (**B**) fed cirrhotic adult patients.

The considerations applied to generate the simulation conditions along with the rationale are summarized in Table 5. The results of the simulations are depicted in Figure 2 and Figure 3. Figure 2A,B show the simulation of various dosing scenarios in non-cirrhotic and cirrhotic pediatric subjects under fasting conditions. Based on the simulations, it was concluded that, under fasting conditions, the AUC with the 0.5 and 1.5 mg/kg doses in all the reference ages (i.e., 2, 6, 12, and 17 years) and in non-cirrhotic and cirrhotic pediatric subjects would be similar to that in non-cirrhotic and cirrhotic adults dosed with the 25 and 100 mg strengths, respectively. Figure 3A,B show the simulation of the dosing scenarios in the same study groups under fed conditions. Based on these simulation results it was concluded that the same doses are still appropriate in all the pediatric groups when SPIR is co-administered with a high-fat, high-calorie meal and that the 1.5 mg/kg dose is safe for cirrhotic subjects as the expected exposures are in line with those predicted in fed adults with liver cirrhosis.

Furthermore, considering the higher bioavailability of the oral suspension, the proposed low (0.5 mg/kg) and high (1.5 mg/kg) doses are in line with the existing treatment guidelines and recommendations for SPIR administration in pediatric subjects with edematous conditions (1–3 mg/kg daily in 1–2 divided doses) [12,13]. In addition, the enrollment of pediatric subjects of any age to receive the higher strength was to be opened only after the confirmation of its safety using the data from the lower strength.

### 3.5. PK Sampling Scheme for a Pediatric PK Trial

**Figure 4:**
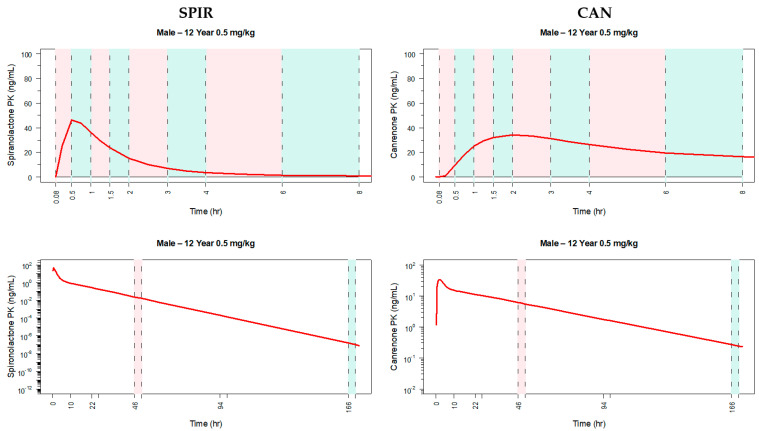
Sampling schedule for pediatric study. (**Top panel**) Plot of predicted spironolactone (**left panel**) and canrenone (**right panel**) PK in a simulated median weight 12-year-old child receiving 0.5.mg/kg of spironolactone where the sampling intervals of sampling subgroups 2.1/3.1 and 2.2/3.2 are superimposed in the same graph. Vertical pink shaded areas represent the sampling intervals of subgroups 2.1/3.1 and vertical green areas represent the sampling intervals of subgroups 2.2/3.2. Plots are truncated at time 8 h for better visualization of the selected times on study day 1. (**Lower panel**) similar plot but showing the sampling times after day 1. Vertical pink lines represent the sampling intervals at 46–50 h while vertical green lines delimited the additional sampling intervals at 166–170 h.

Simulated SPIR and CAN plasma profiles after 0.5 mg/kg of the oral suspension in representative ages were also used to design the PK sampling scheme for the pediatric PK/PD study. As depicted in Table 6, the clinical PK study is to be composed of three population groups depending on age. Group 1 (12 to <17 years): Blood samples plan to be limited to 6 per subject, taken over a period of 7 days (day 1 to day 8 of study). In order to obtain the maximum amount of information from the minimum number of samples per patient, pediatric subjects will be randomized 1:1 into one of two PK sampling subgroups (1.1 and 1.2). These subgroups will require different sampling schedules from time 0 to 8 h post-dose. For both subgroups, 4 samples will be taken per child during the first 8 h post-dose at different sampling intervals, and then 2 additional samples will be taken during days 2 to 8. Group 2 (6 to <12 years) and Group 3 (2 to <6 years): Blood samples will be limited to 5 per subject, taken over a period of 7 days (day 1 to day 8 of study). In order to obtain the maximum amount of information from the minimum number of samples per patient, subjects will be randomized 1:1 into one of two PK sampling subgroups (2.1 and 2.2 for Group 2, and 3.1 and 3.2 for Group 3, respectively). These subgroups will require different sampling schedules from time 0 to 8 h post-dose. For both subgroups, 3 samples per child will be taken during the first 8 h post-dose at different sampling intervals, and then 2 additional samples will be taken during days 2 to 8. A graphical representation of the proposed sampling times per sampling subgroup is presented in Figure 4 for both SPIR and CAN (left and right panels, respectively), and on study day 1 and days 2 to 8 (top and bottom panels, respectively). Only the simulation for 12-year-old children is shown as an example. The simulations were performed to select sampling times that adequately describe SPIR (left) and CAN (right) plasma profiles. Considering the high variability in SPIR absorption and CAN formation phases, a richer sampling time was proposed in this region of the curve. Initially, an identical sampling design is proposed for both the low- and high-dose groups, although the design for the high-dose group will be confirmed or redefined, if needed, with the data collected from the low-dose group.

The results of the evaluation of the study design via simulation/estimation is presented in Section S3 of the Supplementary Materials. The structural parameter estimates obtained with the sparse sampling were similar to those from the original model and the RSE values were less than 35% for all the parameters supporting the suitability of the proposed study design to characterize the PK of SPIR and CAN in pediatrics.

## 4. Discussion

Since the release of the two federal laws the Best Pharmaceuticals for Children Act (BPCA) and the Pediatric Research Equity Act (PREA), more than 500 labeling changes have been made in an attempt to guide and rationalize the administration of drugs in the pediatric population [28]. However, for many drugs, dosing is still largely empirical as there is no information on pediatric dose recommendations on the drug label, resulting in significant off-label use. In addition, the lack of adequate drug formulations, specifically developed for use in pediatrics, worsens the situation. Several problems are associated with the use of adult dosage forms in pediatric subjects, including the use of magistral and officinal preparations, with limited information about accuracy of concentrations from batch to batch, stability, and the safety of excipients found in the adult formulation, which may not be suitable for use in pediatric subjects. The availability of age-appropriate pediatric dosage forms is essential to improve therapeutic outcomes in the pediatric population. SPIR has been used off-label in the pediatric population for more than 60 years; however, there are insufficient studies aimed to inform its clinical efficacy and safety, and no dedicated commercial formulation is currently available for this population. SPIR is currently administered to pediatrics by either breaking the solid dose formulation or by dissolving the solid dose formulation in a liquid vehicle as an extemporaneous preparation. In both cases, once the integrity of the original formulation is altered, the dosing accuracy becomes questionable. Under the above considerations, CMP Pharma developed CaroSpir^®^, an oral suspension formulation of SPIR aimed to improve dosing accuracy and consistency. The clinical development of the oral suspension in adults was based on two relative bioavailability and one food effect study (Table 1) to bridge the information of RLD Aldactone^®^ tablets to the oral suspension. In these studies, the oral suspension resulted in 15% to 37% higher exposures compared to Aldactone^®^ tablets. As the information about the dose proportionality of the oral suspension is limited, and based on the results of studies comparing the suspension to tablets, doses of the suspension higher than 100 mg might result in SPIR concentrations higher than expected. As such, the oral suspension is only approved and marketed for adults with those indications requiring doses of 100 mg or lower.

Based on the above statements, the purpose of CaroSpir^®^ pediatric development is to characterize the PK, safety, tolerability, and pharmacodynamics of single and multiple doses of the SPIR oral suspension in pediatric subjects from birth to <17 years of age with edema due to heart failure or liver cirrhosis. The work presented here corresponds to the model-based optimal design of the pediatric PK trial for these two target indications and in pediatric subjects from 2 to <17 years. As a first step, a parent drug-metabolite popPK model was successfully developed for SPIR and CAN, which was then extrapolated to pediatrics using allometric scaling principles. The decision to use allometric scaling for the extrapolation was based on the fact that the main enzymes involved in SPIR and CAN metabolism remain understudied. It was considered that by the age of 2 years (youngest target age for initial clinical studies) the main enzymes involved in the metabolism were already mature, and the main differences adequately correlate with body weight [24,25,26]. During the evaluation of covariates as part of the adult model development, body weight was not statistically significant for the BSV of clearance or volumes of distribution. However, body weight distribution in the adult clinical studies was narrow with a mean ± SD of 64.08 ± 6.43 kg. Therefore, the fact that body weight was not statistically significant in this specific set of data was not surprising, and a general statement on the effect of body weight on the PK parameters of SPIR/CAN cannot be made with this specific dataset. Moreover, there is substantial empirical evidence of the suitability for the ¾ power allometric model to predict drug clearance and 1 for volumes of distribution. The exponent of ¾ might be used for substances that are eliminated mainly by metabolism, or by metabolism and renal excretion combined, as it is in the case of SPIR and CAN [20,29,30]. In addition, several publications demonstrated the use of allometry as an approach to avoid underestimation of clearance in younger subjects [31,32,33,34]. The model was used to support the selection of appropriate low and high doses of the oral suspension to be used in a single and multiple dose PK/PD study in children and adolescents. Considering the herogeneity of the pediatric subjects to be included in the study, as well as the known impact of food on drug exposure, additional evaluations were performed accounting for PK changes in these settings. Furthermore, the model was used to optimally design a PK sampling strategy in pediatric subjects using simulations.

Limited sampling studies are becoming more common. They are attractive because of their ability to determine important clinical PK information accurately and without bias, while providing convenient schedules with minimal blood draws and reduced load on clinical laboratories. These issues become even more important in pediatrics where, in many cases, limited sampling PK studies are the only option. In this particular case, a good set of prior data (informatively sampled data from a statistically reasonably sized population of subjects) is available with data from 92 healthy adult subjects receiving two different doses of the oral suspension. Despite the available pediatric guidelines and adult data on SPIR dosing, target efficacious exposures have not been well characterized in the adult patient population. This is presumably because Aldactone^®^ tablets have been approved and administered for many years. On its label, clinical exposures in adult patients are not reported (only exposures in healthy adults are included). Additionally, clinical exposure data was not collected in adult patients using the SPIR oral suspension formulation. Therefore, target efficacious exposures for the oral suspension in the pediatric population were initially based on existing data from three phase I studies in healthy subjects (Table 1). The simulation study with the allometricaly scaled popPK model suggested that 0.5 mg/kg and 1.5 mg/kg doses in 2 to <17-year subjects would be equivalent to 25 and 100 mg doses in healthy adults. In addition, in order to evaluate the higher exposures that are anticipated in fed pediatric subjects as well as in subjects with liver cirrhosis, additional simulations were performed accounting for the altered PK of the drug in these clinical scenarios. Specifically, in the case of cirrhotic patients, a reduced SPIR clearance from the central compartment (i.e., CL) by ~85%, metabolic clearance (i.e., CLM1) by ~38%, and CAN clearance from the central compartment (i.e., CLM) by ~72% were considered to reproduce the much longer half-lives observed in adult cirrhotic subjects [17] and may be considered to reproduce the same magnitude of changes in pediatric subjects (additional information provided in the Supplementary Materials on the considerations used to simulate the scenarios in adult patients with liver cirrhosis). In addition, a sensitivity analysis was performed with the adult popPK model to find the changes in the PK parameters needed to achieve the exposures observed in study 084-15 under fed conditions. This analysis revealed that food produces a nearly 94% reduction in SPIR’s ka, leading to the lower Cmax observed in study 084-15. The lower Cmax and larger Tmax observed in the fed state could be due to a slower gastric emptying time with food intake [35]. As a consequence of this decrease in ka, absorption becomes the rate-limiting step and elimination rate gets confounded by absorption, i.e., flip-flop kinetic is expected when SPIR is co-administered with a high-fat, high-calorie meal. This finding is not unexpected and has also been previously reported with other low solubility drugs [36]. In addition, bioavailability is doubled. This could be due to an increased luminal solubilization of SPIR underpinned by the release of bile acids and phospholipids that are stimulated by the lipids from the high-fat food, leading to the formation of different colloidal phases that could potentially solubilize SPIR [37]. Finally, the fraction metabolized to CAN decreased by almost ~60%, probably due to saturation of the metabolic pathway (not completely known) that leads to CAN formation and/or an increased splanchnic blood flow [8,9,10]. These changes were also considered to evaluate the effect of food in the pediatric population (additional information is provided in the Supplementary Materials). Although in these specific scenarios the predicted exposures after the 1.5 mg/kg dose are higher than the exposures observed in fasting healthy adults in the clinical trials, they are within the expected values for adult subjects under the same conditions, i.e., cirrhotic and non-cirrhotic subjects in a fed state. This dose was thus also considered safe for pediatric subjects.

Considering the higher bioavailability of the oral suspension when compared to the tablet, the proposed low (0.5 mg/kg) and high (1.5 mg/kg) doses are in line with the existing scientific literature, guidelines, and recommendations for SPIR administration in pediatric subjects with edematous conditions (1 to 3 mg/kg daily in 1 to 2 divided doses [3,4,38,39,40]). Based on the simulations, these doses were also considered appropriate in pediatric subjects receiving the oral suspension concomitantly with food and/or in patients with liver cirrhosis. Furthermore, the single- and multiple-dose pediatric PK/PD clinical study will administer the lower dose strength first in all age groups, and following acceptable evaluation of PK, PD, safety and tolerability, subsequently proceed with the higher dose strength. All above considerations used to simulate the pediatric scenarios (including allometric scaling, effect of food, and cirrhosis) will be tested once data from actual pediatric subjects from the clinical study become available. The model was also applied via Monte Carlo simulations to propose sampling intervals for the pediatric PK clinical study in three different age groups (Group 1: 12 to <17 years; Group 2: 6 to <12 years; Group 3: ≥2 to <6 years). The simulated profiles were used to establish a range of time to reach maximum plasma concentrations to guide the sampling schedule around the expected peak (where a higher variability was expected based on the PK information in adults). The selection of the sampling times took into consideration that, to completely describe a pharmacokinetic profile, one must obtain a sufficient number of blood samples in the absorption, distribution, and elimination phases. Initially, an identical sampling design is proposed for both the low- and high-dose groups, although the design for the high-dose group will be confirmed or redefined, if needed, based on the data collected from the low-dose group.

## 5. Conclusions

The application of model-based approaches in pediatric drug development provides the means to test different dosing scenarios and evaluate possible safety concerns under specific circumstances. In this specific case of SPIR, a model-based approach helped in the selection of a low and high dose for a pediatric clinical trial in patients with cirrhosis and non-cirrhotic pediatric subjects of different ages, both in fasted and fed states. Moreover, it was used to delineate the sampling times for the PK trial. Even when there are unknowns in the mechanisms behind a drug’s PK, as it is in the case of SPIR, this integrative approach provides a more rational dose selection and improves the design of the clinical study when compared to conventional approaches. All the considerations used for modeling and simulation will be thoroughly evaluated once data from the pediatric subjects involved in the clinical study become available.

## Figures and Tables

**Table 1:** Summary of the studies that were part of CaroSpir’s^®^ clinical development.

Study	Description	Reference Listed Drug	N
063-15 (Pilot)	An open-label, randomized, two-treatment, two-period, two-sequence, crossover, single-dose, oral pharmacokinetic and comparative bioavailability study in healthy, human subjects under fasting conditions	25 mg Aldactone^®^ Tablets	14
064-15 (Pivotal)	An open-label, randomized, two-treatment, two-period, two-sequence, crossover, single-dose, oral pharmacokinetic and comparative bioavailability study in healthy, human adult subjects under fasting conditions	100 mg Aldactone^®^ Tablets	56
084-15 (Food effect)	An open-label, balanced, randomized, single-dose, two-treatment (fed vs. fasting), two-period, two-way cross over, oral food effect study in healthy human adult subjects	100 mg Aldactone^®^ Tablets	23

**Table 2:** Demographic profile of subjects included in studies 063-15, 064-115 and 084-15.

Demographic	063-15	064-15	084-15	All Subjects
	(N = 14)	(N = 56)	(N = 24)	(N = 94)
Age (years) Mean ± SD (median, range)	36.1 ± 4.3 (36.5, 31–43)	29.2 ± 5.5 (28.0, 19–40)	29.5 ± 7.1 (29.0, 20–44)	30.3 ± 6.2 (29, 19–44)
Height (cm) Mean ± SD (median, range)	163.7 ± 5.3 (163.5, 156–173)	167.8 ± 5.7 (167, 157–183)	168.1 ± 6.5 (168, 156–180)	167.2 ± 6 (166.5, 156–183)
Weight (kg) Mean ± SD (median, range)	63.1 ± 4.5 (62.2, 57.4–72.5)	64.4 ± 6.8 (63.6, 53.1–83.1)	64.1 ± 6.4 (62.8, 55.0–78.1)	64.1 ± 6.4 (63.1, 53.1–83.1)
BMI (kg/m^2^) Mean ± SD (median, range)	23.6 ± 1.4 (24, 19.6–24.8)	22.8 ± 1.7 (23.3, 18.6–24.9)	22.6 ± 1.6 (22.9, 19.9–24.8)	22.9 ± 1.7 (23.3, 18.6–24.9)

**Table 3:** Final POPPK model parameter estimates for adults.

Parameter	Model Results			Bootstrap Results	
	Value	% RSE	% Shrinkage	Mean	95% CI
ALAG1	0.156	0.7	-	0.153	0.14–0.17
ka(1/h)	5.22	1.1	-	4.24	4.0–6.3
CL (L/h)	629	3.3	-	636.2	462.8–794.8
V2 (L)	517	2.0	-	540.3	469.5–563.5
Q (L/h)	89.9	2.2	-	90.7	82.3–97.3
V3 (L)	777	1.9	-	785.5	687.9–866.6
Fm	0.7 FIX	-	-	0.7 FIX	
CLM1 (L/h)	217	4.1	-	228.3	146.8–286.7
CLM (L/h)	17	3.6	-	17.8	11.5–22.2
V4 (L)	189	3.4	-	192.8	130.1–248.7
Q1 (L/h)	60	3.9	-	65.1	39.3–80.7
V5 (L)	448	3.0	-	470.3	296.9–598.2
**Between Subject Variability**					
ka (1/h)	0.9	26.2	7	0.85	0.49–1.38
CL (L/h)	0.166	19.8	5	0.18	0.07–0.26
V2 (L)	0.118	23.2	10	0.112	0.07–0.15
CL, V2 (covariance)	0.112	0.02	-	0.114	0.06–0.16
Q (L/h)	0.08	29.1	20	0.08	0.03–0.12
CLM1 (L/h)	0.18	26.7	5	0.17	0.12–0.24
CLM (L/h)	0.08	25.9	12	0.07	0.04–0.11
V4 (L)	0.03	44.9	36	0.02	0.001–0.059
Q1 (L/h)	0.07	35	26	0.1	−0.08–0.22
V5 (L)	0.09	23.1	19	0.08	0.04–0.12
**Residual Error**					
EPS1	0.08	5.5	7	0.08	0.07–0.09
EPS2	0.017	2.5	10	0.017	0.014–0.020

The mean and 95% CIs were generated from a bootstrap run of 1000 resampled datasets, including runs with successful minimization and failed $COV steps. CI, confidence interval; CL, apparent clearance of SPIR from central compartment; CLM1, apparent metabolic clearance of SPIR to CAN; Fm, fraction metabolized from SPIR to CAN; h, hour; L, liter; ALAG1, lag time; ka, absorption rate constant; Q, apparent intercompartmental clearance of SPIR; RSE, relative standard error; V2, apparent central volume of distribution of SPIR; V3, peripheral volume of distribution of SPIR; CLM, apparent clearance of CAN from central compartment; V4, apparent central volume of distribution of CAN; V5, apparent peripheral volume of distribution of CAN; Q1, apparent intercompartmental clearance of CAN; EPS1, proportional residual error for SPIR; EPS2, proportional residual error for CAN.

**Table 4:** Parameter values extrapolated to various pediatric age groups using allometric scaling.

Age Group	CL	V2	Q	V3	ka	V4	CLM	Q1	V5	ALAG1	Fm	CLM1
	(L/h)	(L)	(L/h)	(L)	(h^−1^)	(L)	(L/h)	(L/h)	(L)	(h)		(L/h)
Adults	629.00	517.00	89.90	777.00	5.22	189.00	17.00	60.00	448.00	0.156	0.70	217.00
2 years—female	174.13	93.28	24.89	140.19	5.22	34.10	4.71	16.61	80.83	0.160	0.70	60.07
2 years—male	165.27	87.00	23.62	130.76	5.22	31.81	4.47	15.76	75.39	0.160	0.70	57.02
6 years—female	260.33	159.46	37.21	239.65	5.22	58.29	7.04	24.83	138.18	0.160	0.70	89.81
6 years—male	259.33	158.65	37.06	238.43	5.22	58.00	7.01	24.74	137.47	0.160	0.70	89.47
12 years—female	433.78	315.00	62.00	473.42	5.22	115.16	11.72	41.38	272.96	0.160	0.70	149.65
12 years—male	439.41	320.47	62.80	481.63	5.22	117.15	11.88	41.92	277.70	0.160	0.70	151.59
17 years—female	589.42	474.09	84.24	712.51	5.22	173.31	15.93	56.22	410.82	0.160	0.70	203.35
17 years—male	522.85	404.07	74.73	607.28	5.22	147.72	14.13	49.87	350.14	0.160	0.70	180.38

CL, apparent clearance of SPIR from central compartment; CLM1, apparent metabolic clearance of SPIR to CAN; Fm, fraction metabolized from SPIR to CAN; h, hour; L, liter; ALAG1, lag time; ka, absorption rate constant; Q, apparent intercompartmental clearance of SPIR; V2, apparent central volume of distribution of SPIR; V3, peripheral volume of distribution of SPIR; CLM, apparent clearance of CAN from central compartment; V4, apparent central volume of distribution of CAN; V5, apparent peripheral volume of distribution of CAN; Q1, apparent intercompartmental clearance of CAN.

**Table 5:** Summary of the considerations applied to generate the simulation conditions for non-cirrhotic and cirrhotic scenarios, along with the rationale.

Condition/State	Parameter Affected	% of Change from the Model Estimate	Rationale	Reference(s)
Non-cirrhotic under fasting conditions	NA	NA	Model parameter estimates are used for the simulations	
Cirrhotic under fasting conditions	CL	↓ 84.44%	Based on the literature	Sungaila et al. [17] Gardiner et al. [23]
	CLM1	↓ 37.76%	Reduced to achieve T_max_ for CAN in literature	Sungaila et al. [17] Gardiner et al. [23]
	CLM	↓ 71.55%	Based on the literature	Sungaila et al. [17] Gardiner et al. [23]
Non-cirrhotic under fed conditions	f	↑ 100%	Based on fed data	Study 8415
	ka	↓ 93.5%	Based on fed data	Study 8415
	k	changed to new ka	K(original) >> ka, hence a flip-flop PK was considered which matches to the profile in fed data	Study 8415
	Fm	↓ 60%	Based on fed data	Study 8415
Cirrhotic under fed conditions	f	↑ 100%	Based on fed data	Study 8415
	ka	↓ 93.5%	Based on fed data	Study 8415
	CL	↓ 84.44%	Based on the literature	Sungaila et al. [17] Gardiner et al. [23]
	Fm	↓ 60%	Based on fed data	Study 8415
	CLM1	↓ 37.76%	Reduced to achieve T_max_ for CAN in literaure	Sungaila et al. [17] Gardiner et al. [23]
	CLM	↓ 71.55%	Based on the literature	Sungaila et al. [17] Gardiner et al. [23]

CL, apparent clearance of SPIR from central compartment; CLM1, apparent metabolic clearance of SPIR to CAN; CLM, apparent clearance of CAN from central compartment; f, factor for the change in the bioavailability of SPIR; ka, absorption rate constant; Fm, fraction metabolized from SPIR to CAN; T_max_, Time to reach C_max_ (maximum concentrations observed in the central compartment); k (=CL/V2), elimination rate constant for SPIR central compartment; NA, not applicable as this scenarios is represented by the study data and POPPK model; flip-flop PK is a phenomenon that arise when ka is much lower than k, where elimination of the drug is governed by ka.

**Table 6:** PK study design for Group 1 (12 to <17 years) and Groups 2–3 (6 to <12 years and 2 to <6 years, respectively).

Group 1 (12 to <17 years) (Total N = 6)		
Subgroups (N = Number of Subjects)	Sampling Intervals after Dose Administration	Number of Samples per Subject
Subgroup 1.1 (N = 3)	5–30 min (0.08–0.5 h) 1–1.5 h 2–3 h 4–6 h	4
Subgroup 1.2 (N = 3)	30 min–1 h 1.5–2 h 3–4 h 6–8 h	4
1.1, 1.2 (N = 6)	46–50 h	1
1.1, 1.2 (N = 6)	166–170 h	1
**Group 2–3 (2 to <12 years) (Total N = 6 per group)**		
**Subgroups (N = Number of Subjects)**	**Sampling Intervals after Dose Administration**	**Number of Samples per Subject**
Subgroups 2.1 (N=3), 3.1 (N = 3)	5–45 min (0.08–0.75 h) 1.5–2.5 h 3.5–6 h	3
Subgroups 2.2 (N=3), 3.2 (N = 3)	45 min–1.5 h (0.75–1.5 h) 2.5–3.5 h 6–8 h	3
2 (N=6), 3 (N = 6)	46–50 h	1
2 (N=6), 3 (N = 6)	166–170 h	1

Subgroups defined by age within subgroups 1.1 and 1.2 (12 to <17 years), 2.1 and 2.2 (≥6 to <12 years), and 3.1 and 3.2 (≥2 to <6 years).

## Data Availability

The data that support the findings of this study are available from the corresponding author, [P.M], upon reasonable request.

## Supplementary Materials

The following are available online at https://www.mdpi.com/article/10.3390/pharmaceutics13060849/s1, Figure S1: DV versus PRED (left panel) and DV versus IPRED plots for SPIR from the parent-metabolite model. Black: 25 mg dose; Blue: 100 mg dose, Figure S2: DV versus PRED (left panel) and DV versus IPRED plots for CAN from the parent-metabolite model. Black: 25 mg dose; Blue: 100 mg dose, Figure S3: Diagnostics plots for SPIR from the parent-metabolite model: CWRES vs. PRED (left panel) and CWRES vs. Time (right panel). Black: 25 mg dose; Blue: 100 mg dose, Figure S4: Diagnostics plots for CAN from the parent-metabolite model: CWRES vs. PRED (left panel) and CWRES vs. Time (right panel). Black: 25 mg dose; Blue: 100 mg dose, Figure S5: Diagnostics plots for SPIR from the parent-metabolite model: IWRES vs. PRED (left panel) and IWRES vs. Time (right panel). Black: 25 mg dose; Blue: 100 mg dose, Figure S6: Diagnostics plots for CAN from the parent-metabolite model: IWRES vs. PRED (left panel) and IWRES vs. Time (right panel). Black: 25 mg dose; Blue: 100 mg dose, Figure S7: Observed (DV), individual predicted (IPRED) and population predicted (PRED) concentrations versus time estimated from the final model of SPIR, Figure S8: Observed (DV), individual predicted (IPRED) and population predicted (PRED) concentrations versus time estimated from the final model of CAN, Figure S9: Relationship between ETAs and covariates (a) demographics and (b) clinical covariates, Figure S10: Visual predictive check for SPIR (left panel) and CAN (right panel) after the 25 mg dose (top panel) and 100 mg dose (bottom panel). FLAG1 = 1 and 3: SPIR 25 mg and 100 mg, respectively; FLAG1 = 2 and 4: CAN 25 mg and 100 mg, respectively, Figure S11: Comparison of the PK metrics from model A (original model) and B (sparse sampling model). Panel 1: AUC of SPIR; 2: AUC of CAN; 3: Cmax of SPIR; 4: Cmax of CAN. The green diamonds and solid blue lines reflect the mean and (±) SD of the different PK metrics in adults and 2 years old children after different doses with model A. The orange diamonds and solid purple lines reflect the mean and (±) SD of the different PK metrics in adults and 2 years old children after different doses with model B, Table S1: Literature-informed changes in spironolactone and canrenone clearance to reproduce the changes in the pharmacokinetic metrics in adult patients with liver cirrhosis, Table S2: Comparison between various kinetic parameters in fasting and fed conditions in study 084-15, Table S3: Sensitivity analysis based parameter values that optimized to produce the changes with food effect on SPIR PK in adults, Table S4: Rate constants applied for SPIR and CAN kinetics in fasting and fed conditions in cirrhotic and non-cirrhotic groups, Table S5: Comparison of the parameter estimates from the original and sparse sampling model.
