# Supplementary material for: Model-Informed Optimization of a Pediatric Clinical Pharmacokinetic Trial of a New Spironolactone Liquid Formulation
Source: Pharmaceutics. 2021 Jun 8;13(6):849. doi: 10.3390/pharmaceutics13060849 (PMC8228864; doi:10.3390/pharmaceutics13060849)
Supplement: Supplementary file 1 [file pharmaceutics-13-00849-s001.zip › pharmaceutics-1147370 - sp final.pdf]

pharmaceutics

Supplementary Materials: Model-informed optimization of a pediatric clinical pharmacokinetic trial of a new spironolactone liquid formulation

Manasa Tatipalli, Vijay Kumar Siripuram, Tao Long, Diana Shuster, Galina Bernstein, Pierre Martineau, Kim A. Cook, Rodrigo Cristofoletti, Stephan Schmidt and Valvanera Vozmediano

Section 1: Population pharmacokinetic analysis

The parent- metabolite population pharmacokinetic (popPK) model was done using NONMEM® program (double precision, version 7.3 ICON Development Solutions, Ellicott City, Maryland) with the NMTRAN pre-processor, and the ADVAN routines, mostly ADVAN13 with TOL = 6 for the parent metabolite model and $DES (differential equation) as required. Other supportive software used for data management, graphics, metadata handling and plotting were R® (version 3.6.0 [www.r-project.org] running under Rstudio interface), Pirana v2.9.9 (http://www.pirana-software.com/) and Perl-Speaks-NONMEM v4.9.0 (https://uupharmacometrics.github.io/PsN). Modeling and simulation was done following standard and well-accepted population guidances [1–4].

One, two and three compartment disposition models with first order absorption (ka) were explored. A simultaneous parent-metabolite model was developed for both SPIR and CAN.

In all analyses, the interindividual variability (IIV) was modeled as exponential (e.g., for CL):

$$CL_j = \overline{CL} \times \exp\left(\eta_{jCL}\right) \quad (1)$$

where η_{jCL} denotes the (proportional) difference between the true parameter (CLj) of individual j and the typical value ($\overline{CL}$) in the population. The IIV was modeled the same way for the other parameters. The η's are zero mean random variables with variance ω^2 (e.g., ω^2CL). The ω^2's are the diagonal elements of the inter-individual variance-covariance matrix, Ω. Alternative correlation structures were assumed between the modeled variances (BLOCK option in NONMEM), thus assuming covariance between the η's. The use of an exponential model for the variability prescribes a log-normal distribution for the parameters and then ω is an approximate coefficient of variation. Residual (unexplained) variability in SPIR and CAN plasma concentration, representing a composite of model misspecification, variability in the analytical method, intraindividual variability, digression between the actual and nominal sampling and dosing times, as well as other undefined factors, was modeled as proportional, according to:

$$Cp_{ij} = \widetilde{Cp_{ij}} \times \left(1 + \varepsilon_{ij}\right) \quad (2)$$

where Cp_{ij} and $\widetilde{Cp_{ij}}$ are the ith measured and model predicted (true) concentrations, respectively for patient j and ε_{ij} denotes the residual intra-patient random error, distributed with zero mean and variance σ^2. Other error models were also tested during the model development process.

The individual Bayesian PK parameters estimates were simultaneously obtained with the population mean parameters in the run of the final model.

Analysis of Covariates

The individual PK parameter estimates were plotted against the demographic covariates such as age, body weight (WT), body mass index (BMI) and body surface area (BSA) for visual inspection. In addition, the following clinical laboratory test values were also tested as covariates: serum urea, serum creatinine, serum sodium, serum potassium, total proteins and albumin.

Covariates that showed a visual correlation with a PK parameter were entered into the model for further tests. The selection of covariates used a forward and backward selection process. During forward selection, a covariate was selected only if a significant ($P < 0.05$) decrease (reduction >3.84) in objective function (OFV) from the covariate-free model was obtained. Then all the covariates found to be significant during the forward selection were added simultaneously into a 'full' model. The importance of each covariate was re-evaluated by backward selection. Each covariate was independently removed from the full model to confirm its relevance. An increase in the objective function of >6.635 ($P <$

0.01) was required to confirm that the covariate was significant. The resulting model was called the 'final' population pharmacokinetic model that included all the significant covariates.

Goodness of Fit

The model's goodness of fit to the data was evaluated using the following criteria: 1) obtaining a converging NONMEM run with FOCE-INTER, 2) on the basis of examination of the objective function (OFV = −2 × Log-likelihood value), 3) physiological/pharmacological characteristics of the PK parameters and their variability 4) adequate standard error of estimate for the structural parameters, 5) at least 3 significant digits, 6) estimation of variance-covariance matrix, 7) evaluation of standard diagnostic plots [3–4], and 8) identification of outliers based on the inspection of conditional weighted residuals (CWRES). Outliers were defined as |CWRES| >6 and considered influential when a change of >20% in one or more of the key parameters was observed during sensitivity analysis (running models with and without outliers).

Standard diagnostic plots are presented in Figures S1–S6 below.

Qualification of the Model by Visual Predictive Check

Visual predictive checks (VPC) are based on simulations of model predictions including random effects (especially between-subject variability). A visual comparison of the distribution of the predictions and observations is then performed. VPC was used to internally qualify the developed model for SPIR/CAN in order to verify its precision, stability and predictability. The VPC assesses the influence of variability (IIV and residual) in parameters. Visual predictive check (plot comparing 95% prediction interval with observed data) was performed for the final model generated based on the dataset (SPIR = 92 IDs and 3159 observations and CAN = 92 IDs and 3159 observations). A simulation of N = 1000 was performed based on the structure of the original data, and portrayed as mean and 95%CI. The observed data set was then overlaid. It was expected that the observed mean would fall near the modeled predicted mean and that less than 5% of observations would lie outside the two-sided 95% CI. This comparison was used to evaluate whether the derived model and associated parameters could properly reproduce the observed data.

VPC results are presented in Figure S7 below.

Model Qualification Using Bootstrapping

Bootstrapping was performed to validate the models of all analytes. Once the model was qualified as described above, the robustness of the model and the accuracy of parameter estimates (SE computation) were assessed using a bootstrap method. From the original dataset of 92 patients, 1000 bootstrap sets of 92 individuals were drawn with replacement (re-sampling technique). For each of the 1000 bootstrap sets, the population PK parameters were calculated. With the 1000 estimates of each population PK parameter, the corresponding mean, median, standard deviation, 5th, 50th and 95th percentiles were calculated using PsN. The percentage of successful runs was computed to evaluate the stability of the model. The distribution of the typical value of the main (physiologically relevant: CL/F & Vd/F) population parameters were plotted with the "original" value. The mean parameter estimates obtained from these bootstraps were compared (by computing the difference in percentage) with those obtained in the final model of the original dataset.

The results of the bootstrap is presented in table 3 in the main manuscript.

Goodness of Fit and VPC Plots:

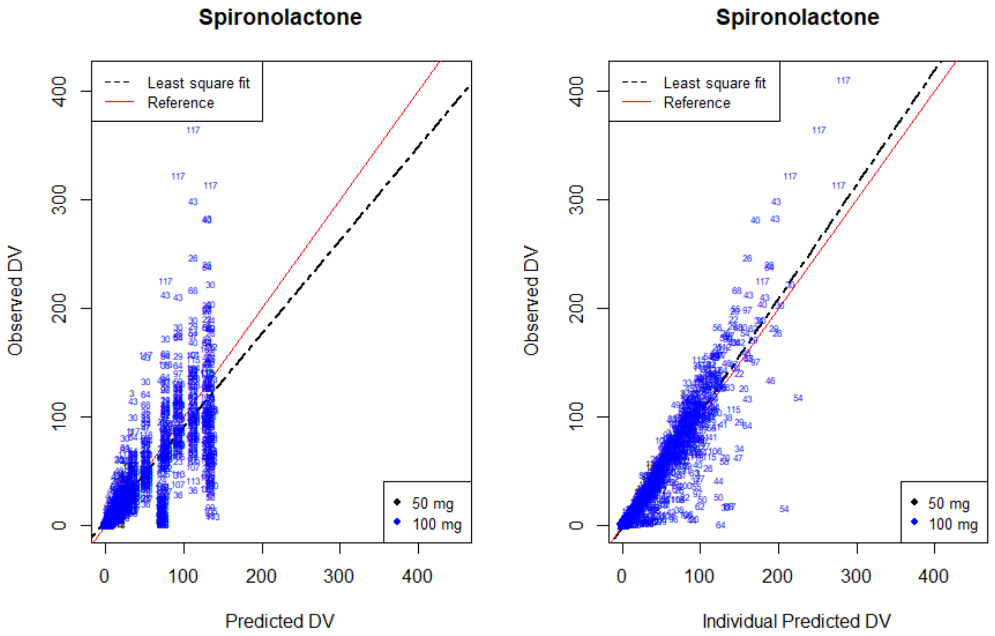

Figure S1. DV versus PRED (left panel) and DV versus IPRED plots for **SPIR** from the parent-metabolite model. Black: 25 mg dose; Blue: 100 mg dose.

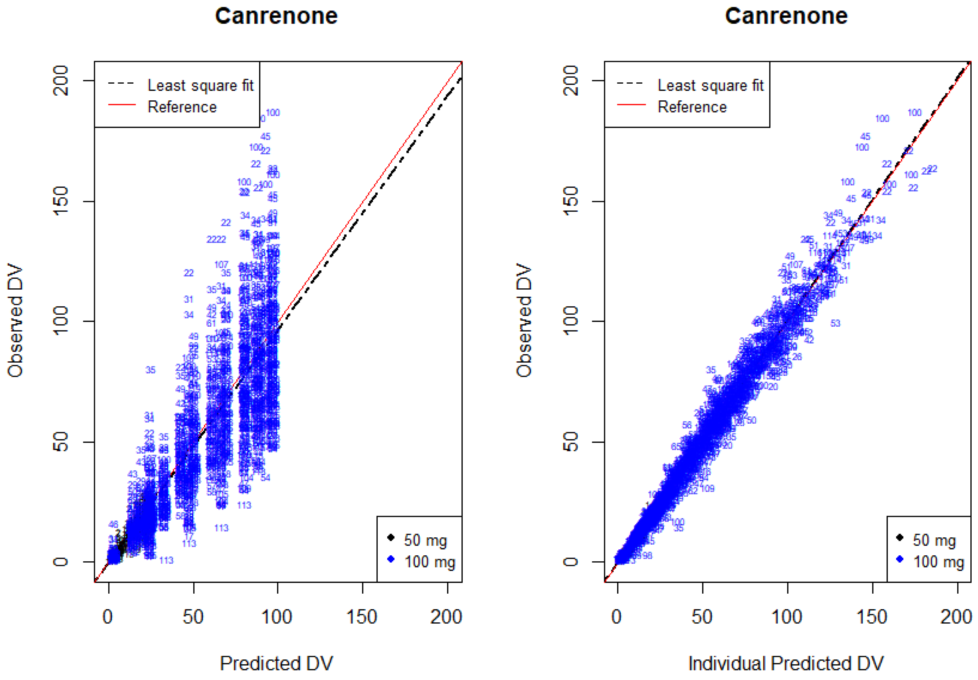

Figure S2. DV versus PRED (left panel) and DV versus IPRED plots for **CAN** from the parent-metabolite model. Black: 25 mg dose; Blue: 100 mg dose.

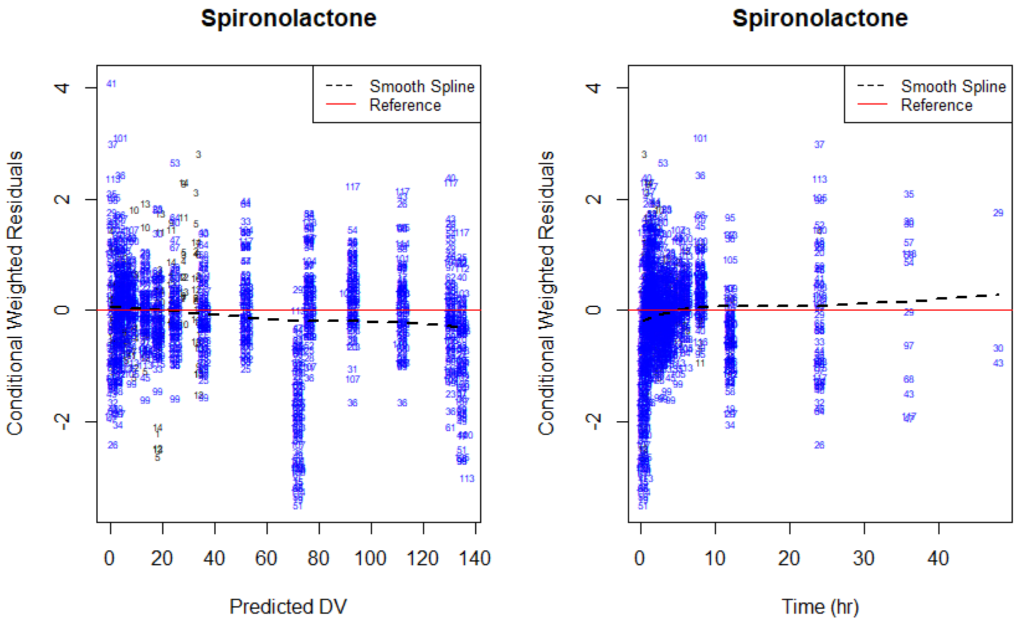

Figure S3. Diagnostics plots for SPIR from the parent-metabolite model: CWRES vs. PRED (left panel) and CWRES vs. Time (right panel). Black: 25 mg dose; Blue: 100 mg dose.

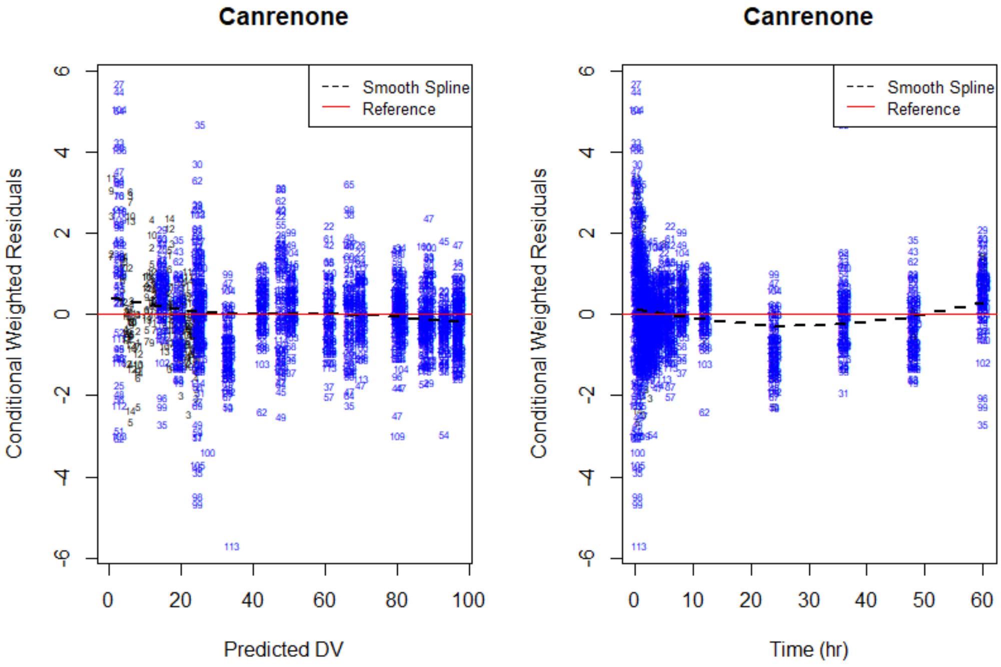

Figure S4. Diagnostics plots for **CAN** from the parent-metabolite model: CWRES vs. PRED (left panel) and CWRES vs. Time (right panel). Black: 25 mg dose; Blue: 100 mg dose.

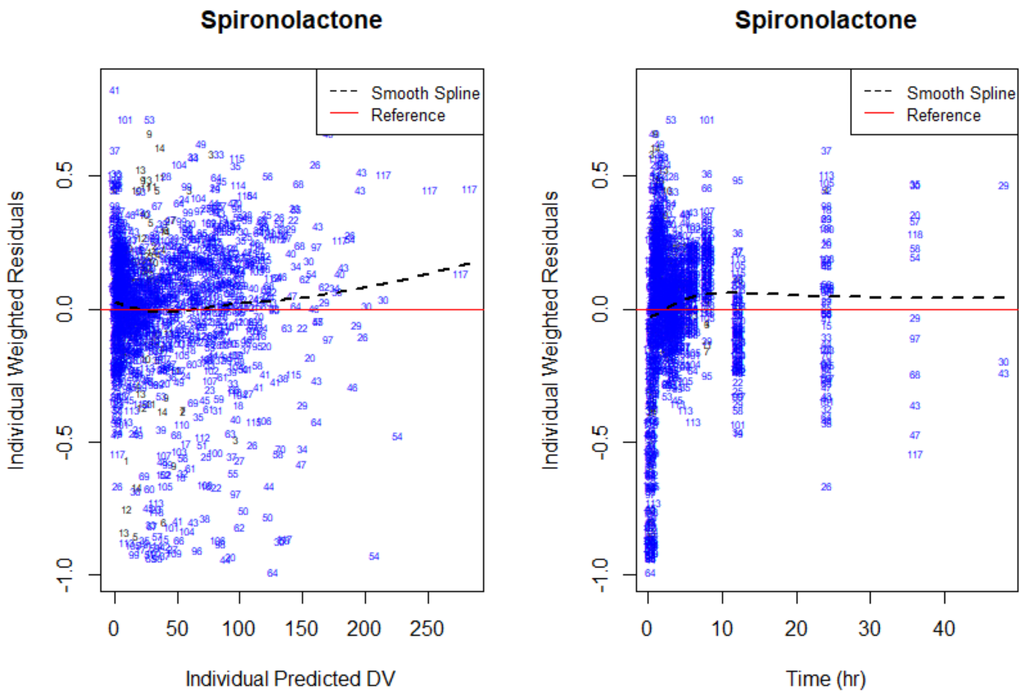

Figure S5. Diagnostics plots for SPIR from the parent-metabolite model: IWRES vs. PRED (left panel) and IWRES vs. Time (right panel). Black: 25 mg dose; Blue: 100 mg dose.

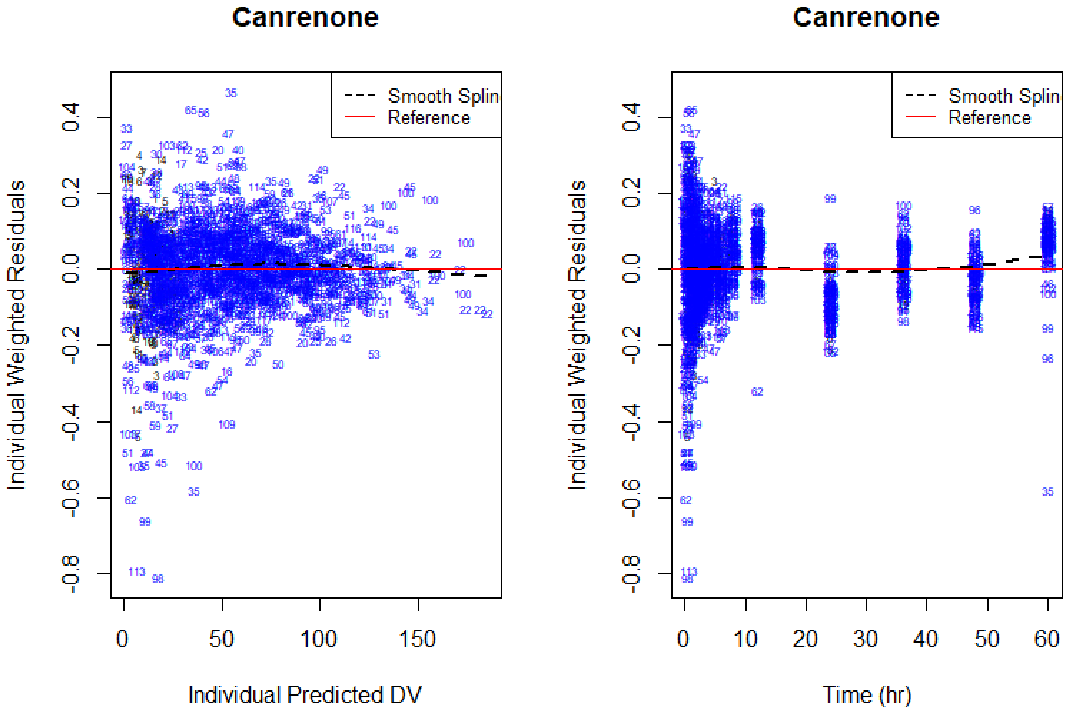

Figure S6. Diagnostics plots for **CAN** from the parent-metabolite model: IWRES vs. PRED (left panel) and IWRES vs. Time (right panel). Black: 25 mg dose; Blue: 100 mg dose.

PopPK Analysis Additional Information

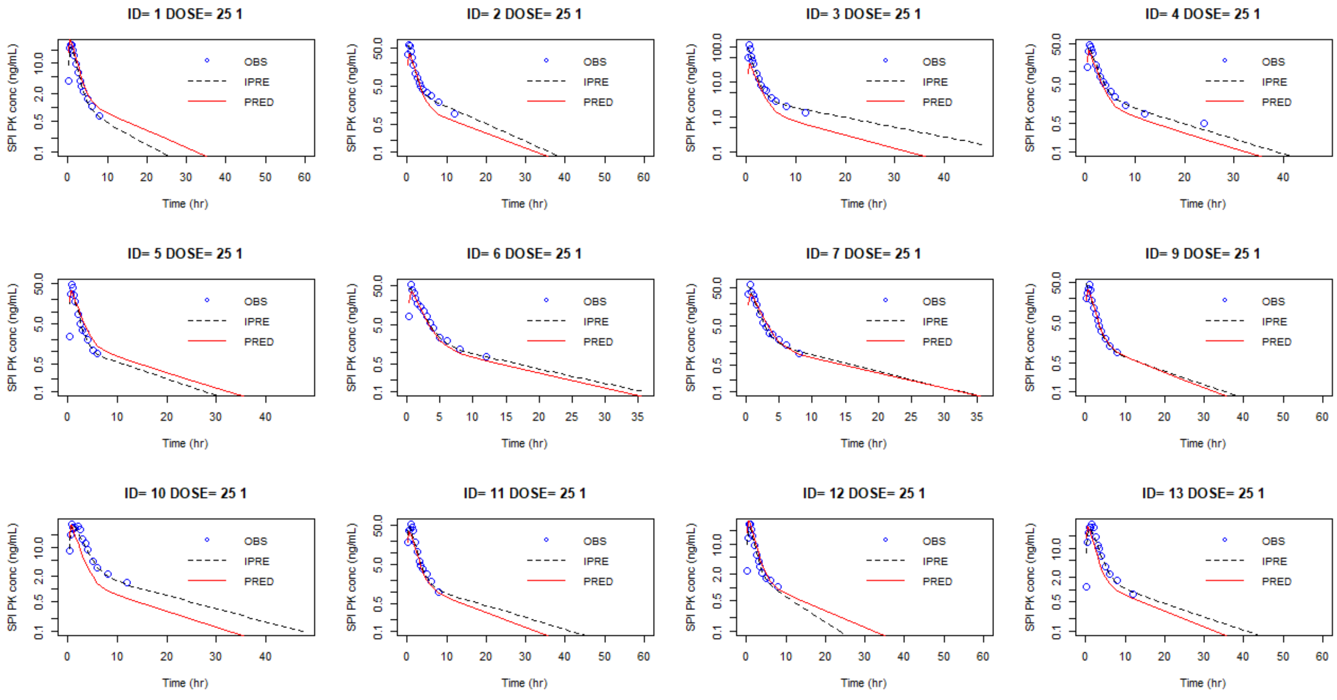

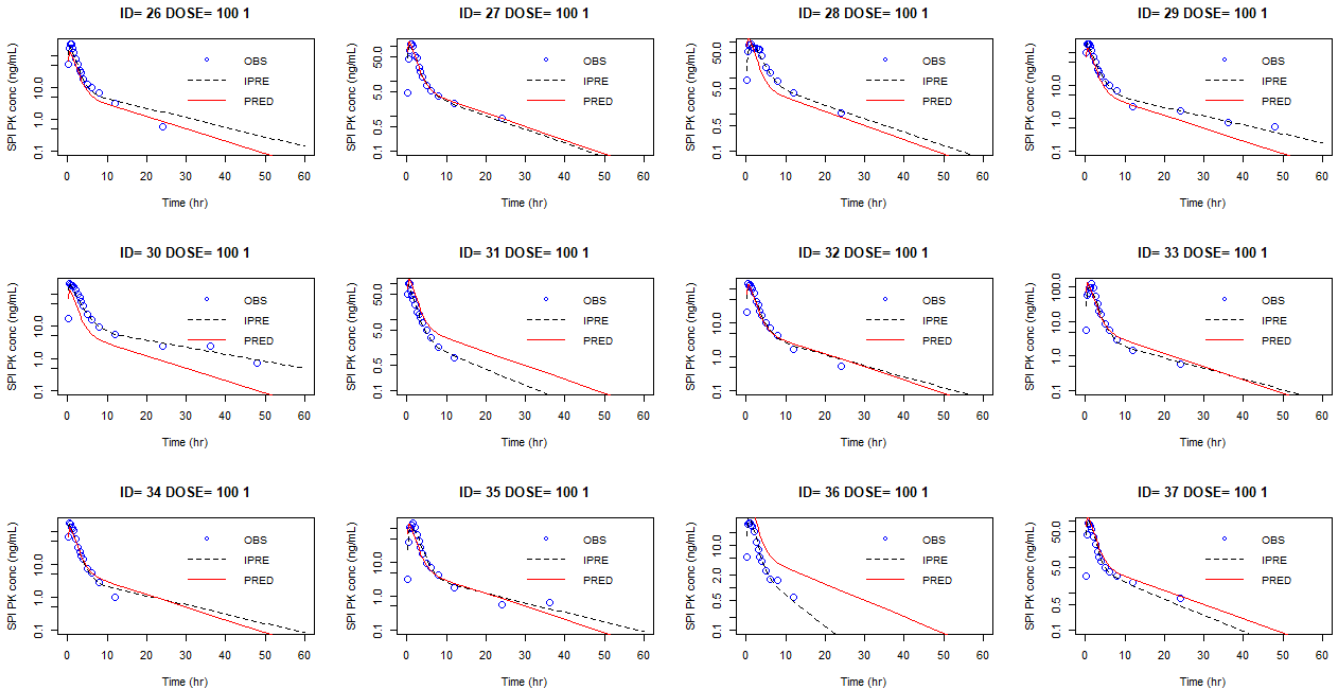

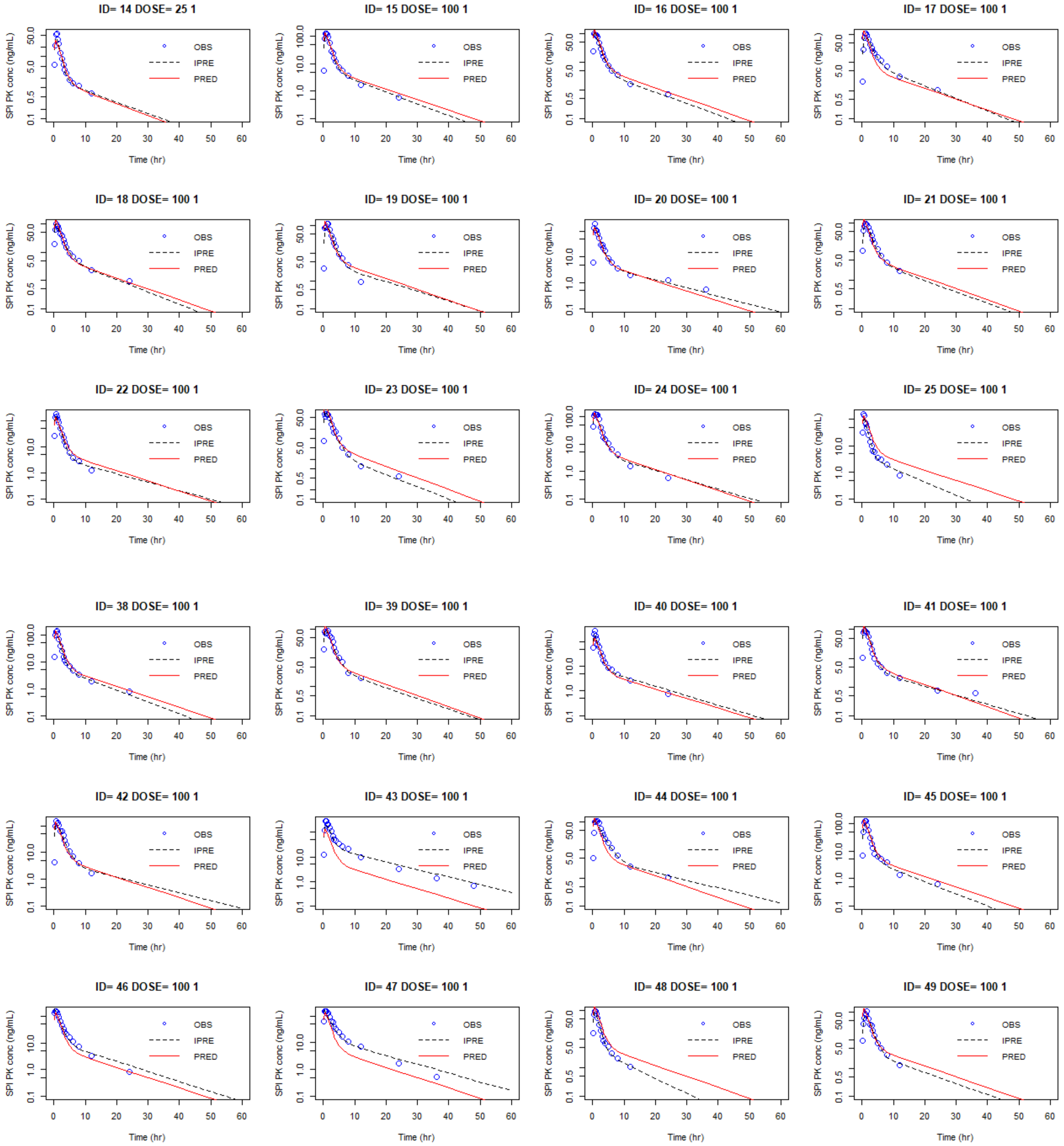
ID= 14 DOSE= 25 1
ID= 15 DOSE= 100 1
ID= 16 DOSE= 100 1
ID= 17 DOSE= 100 1
ID= 18 DOSE= 100 1
ID= 19 DOSE= 100 1
ID= 20 DOSE= 100 1
ID= 21 DOSE= 100 1
ID= 22 DOSE= 100 1
ID= 23 DOSE= 100 1
ID= 24 DOSE= 100 1
ID= 25 DOSE= 100 1
ID= 38 DOSE= 100 1
ID= 39 DOSE= 100 1
ID= 40 DOSE= 100 1
ID= 41 DOSE= 100 1
ID= 42 DOSE= 100 1
ID= 43 DOSE= 100 1
ID= 44 DOSE= 100 1
ID= 45 DOSE= 100 1
ID= 46 DOSE= 100 1
ID= 47 DOSE= 100 1
ID= 48 DOSE= 100 1
ID= 49 DOSE= 100 1
OBS
IPRE
PRED
SPI PK conc (ng/mL)
Time (hr)

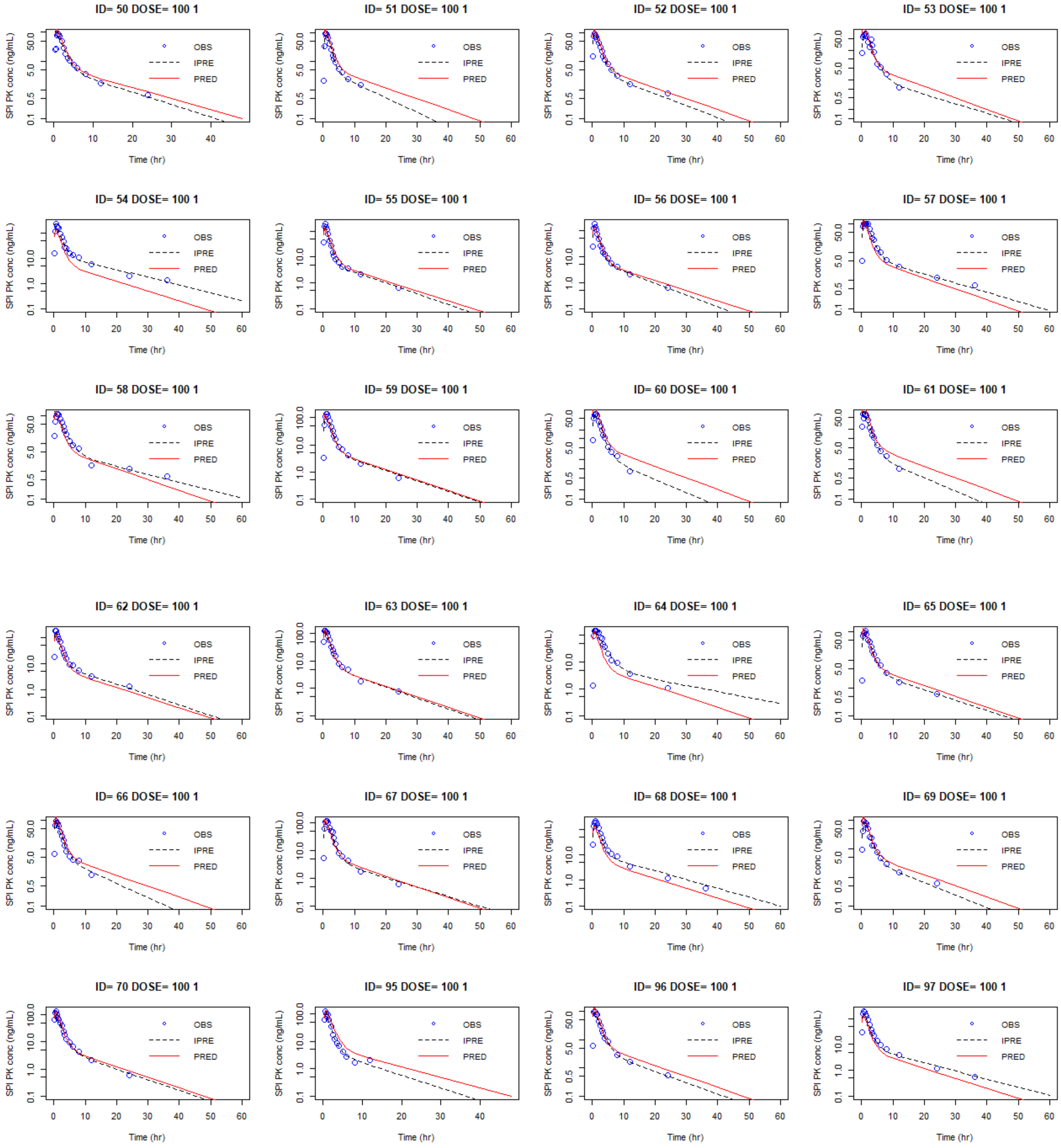
ID= 50 DOSE= 100 1
ID= 51 DOSE= 100 1
ID= 52 DOSE= 100 1
ID= 53 DOSE= 100 1
ID= 54 DOSE= 100 1
ID= 55 DOSE= 100 1
ID= 56 DOSE= 100 1
ID= 57 DOSE= 100 1
ID= 58 DOSE= 100 1
ID= 59 DOSE= 100 1
ID= 60 DOSE= 100 1
ID= 61 DOSE= 100 1
ID= 62 DOSE= 100 1
ID= 63 DOSE= 100 1
ID= 64 DOSE= 100 1
ID= 65 DOSE= 100 1
ID= 66 DOSE= 100 1
ID= 67 DOSE= 100 1
ID= 68 DOSE= 100 1
ID= 69 DOSE= 100 1
ID= 70 DOSE= 100 1
ID= 95 DOSE= 100 1
ID= 96 DOSE= 100 1
ID= 97 DOSE= 100 1
SPI PK conc (ng/mL)
Time (hr)
OBS
IPRE
PRED

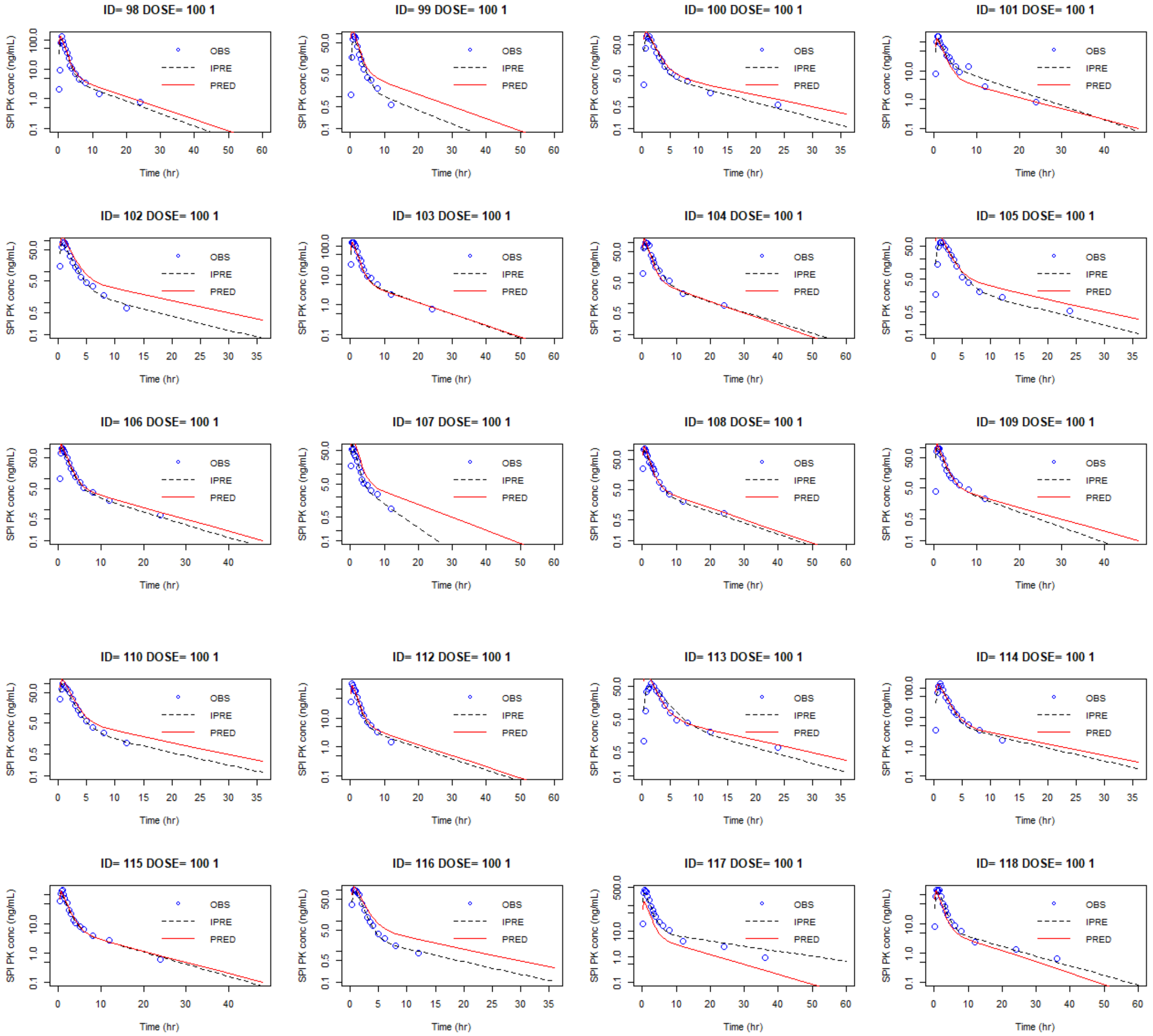

Figure S7. Observed (DV), individual predicted (IPRED) and population predicted (PRED) concentrations versus time estimated from the final model of **SPIR.**

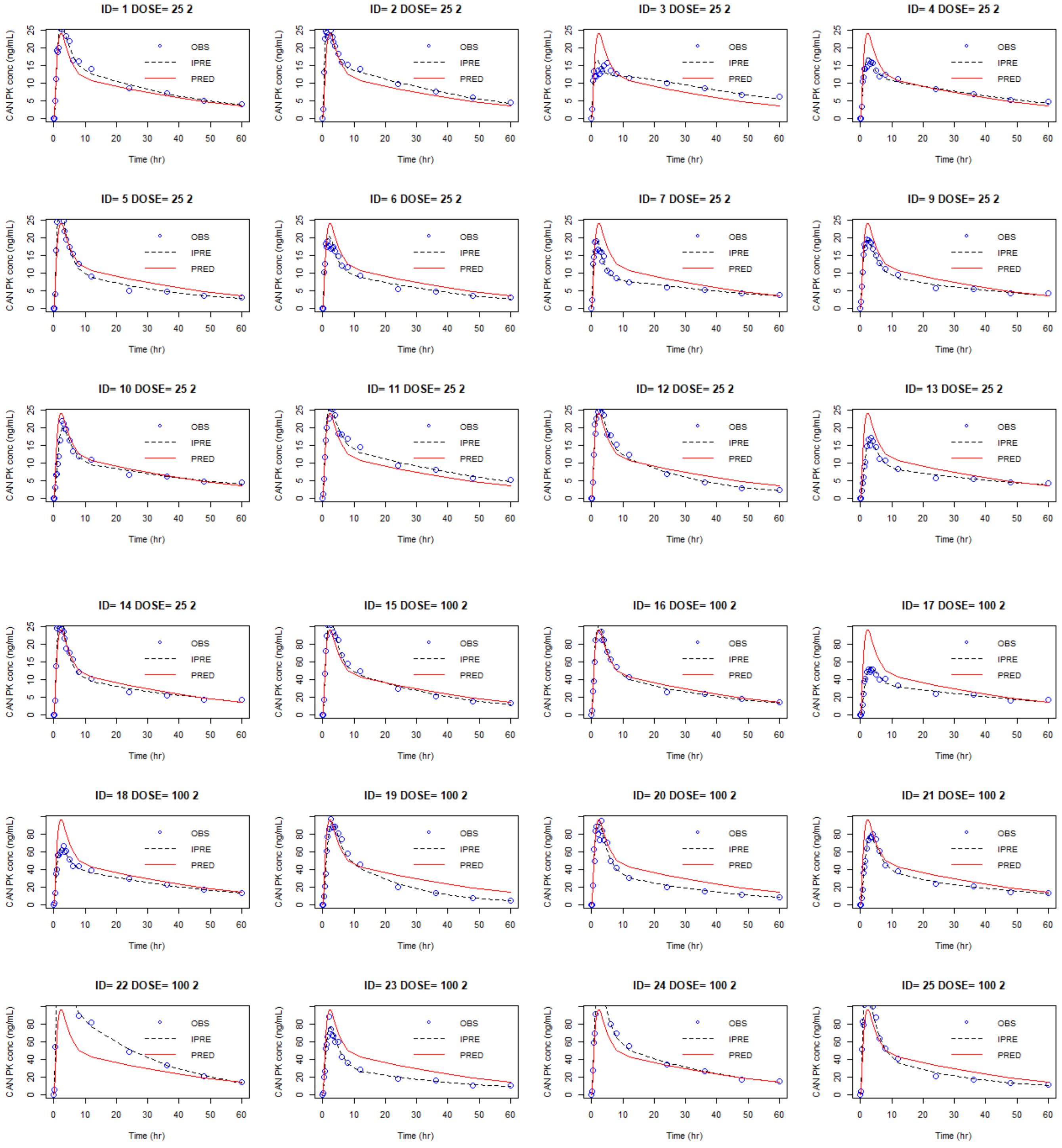
ID= 1 DOSE= 25 2
ID= 2 DOSE= 25 2
ID= 3 DOSE= 25 2
ID= 4 DOSE= 25 2
ID= 5 DOSE= 25 2
ID= 6 DOSE= 25 2
ID= 7 DOSE= 25 2
ID= 9 DOSE= 25 2
ID= 10 DOSE= 25 2
ID= 11 DOSE= 25 2
ID= 12 DOSE= 25 2
ID= 13 DOSE= 25 2
ID= 14 DOSE= 25 2
ID= 15 DOSE= 100 2
ID= 16 DOSE= 100 2
ID= 17 DOSE= 100 2
ID= 18 DOSE= 100 2
ID= 19 DOSE= 100 2
ID= 20 DOSE= 100 2
ID= 21 DOSE= 100 2
ID= 22 DOSE= 100 2
ID= 23 DOSE= 100 2
ID= 24 DOSE= 100 2
ID= 25 DOSE= 100 2
CAN PK conc (ng/mL)
Time (hr)
OBS
IPRE
PRED

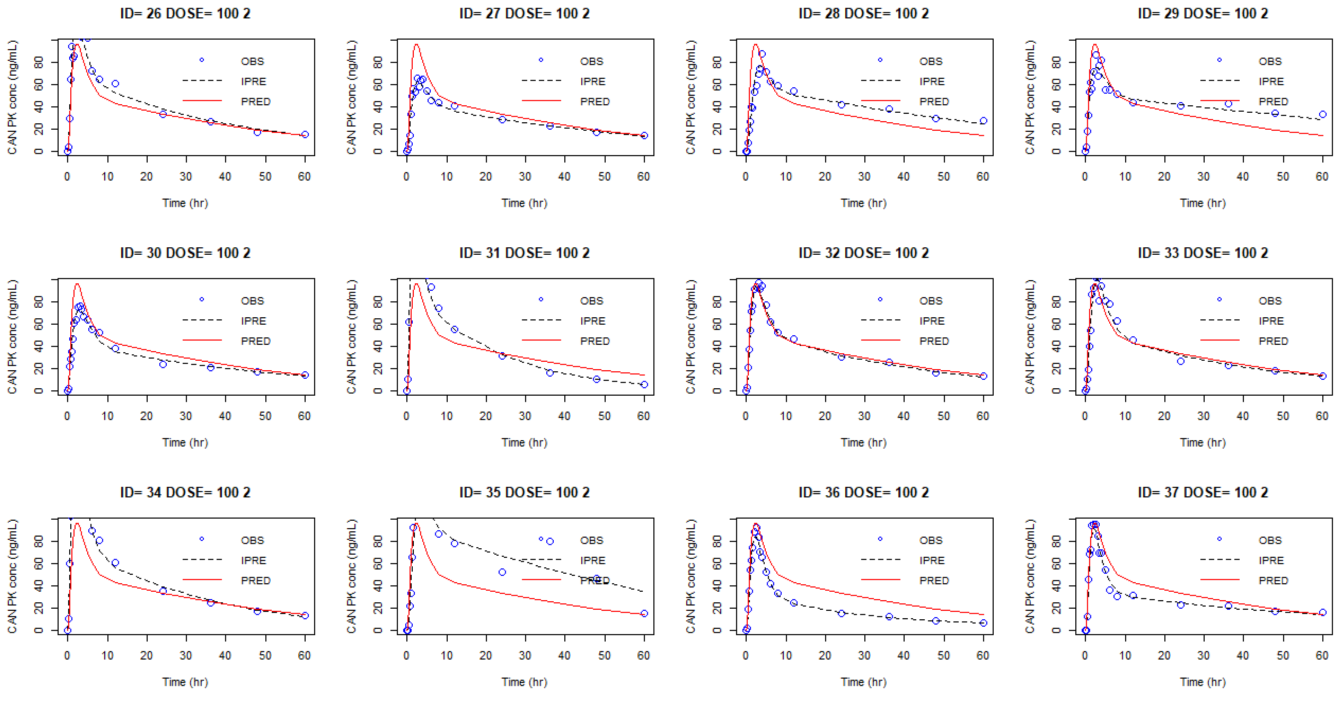
ID= 26 DOSE= 100 2
ID= 27 DOSE= 100 2
ID= 28 DOSE= 100 2
ID= 29 DOSE= 100 2
ID= 30 DOSE= 100 2
ID= 31 DOSE= 100 2
ID= 32 DOSE= 100 2
ID= 33 DOSE= 100 2
ID= 34 DOSE= 100 2
ID= 35 DOSE= 100 2
ID= 36 DOSE= 100 2
ID= 37 DOSE= 100 2
CAN PK conc (ng/mL)
Time (hr)
OBS
IPRE
PRED

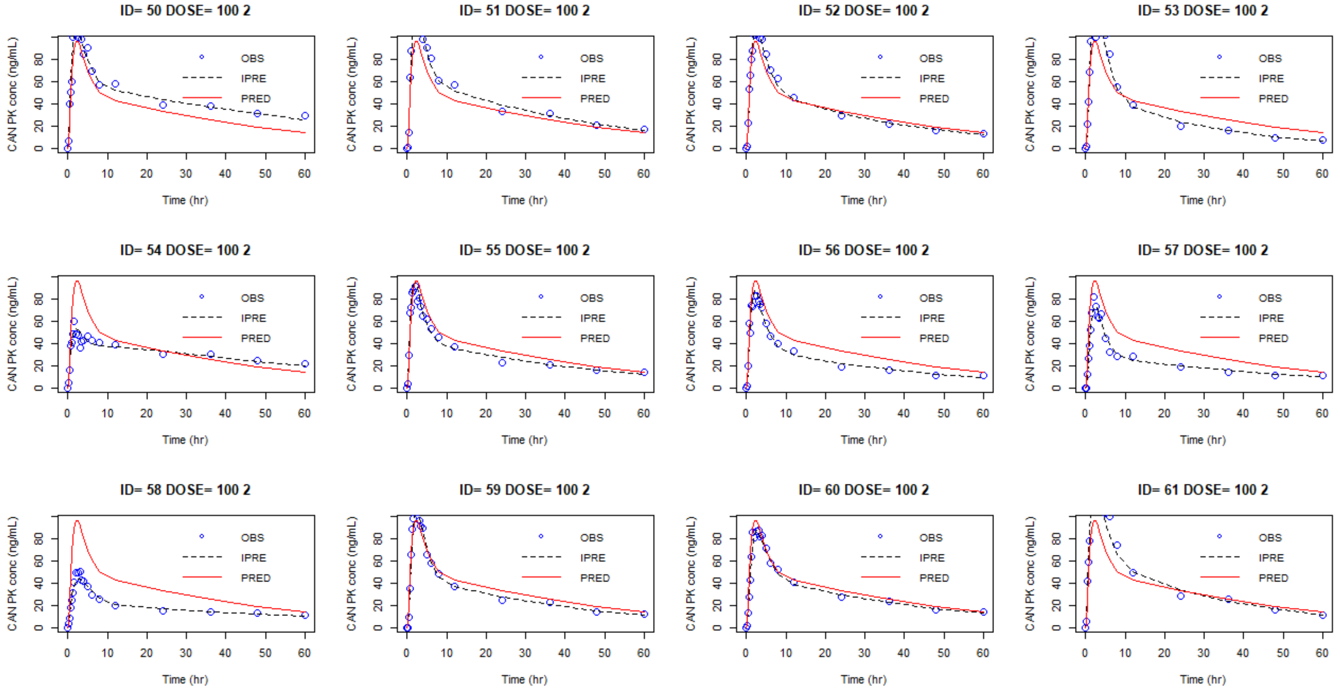
ID= 50 DOSE= 100 2
ID= 51 DOSE= 100 2
ID= 52 DOSE= 100 2
ID= 53 DOSE= 100 2
ID= 54 DOSE= 100 2
ID= 55 DOSE= 100 2
ID= 56 DOSE= 100 2
ID= 57 DOSE= 100 2
ID= 58 DOSE= 100 2
ID= 59 DOSE= 100 2
ID= 60 DOSE= 100 2
ID= 61 DOSE= 100 2
CAN PK conc (ng/mL)
Time (hr)
OBS
IPRE
PRED

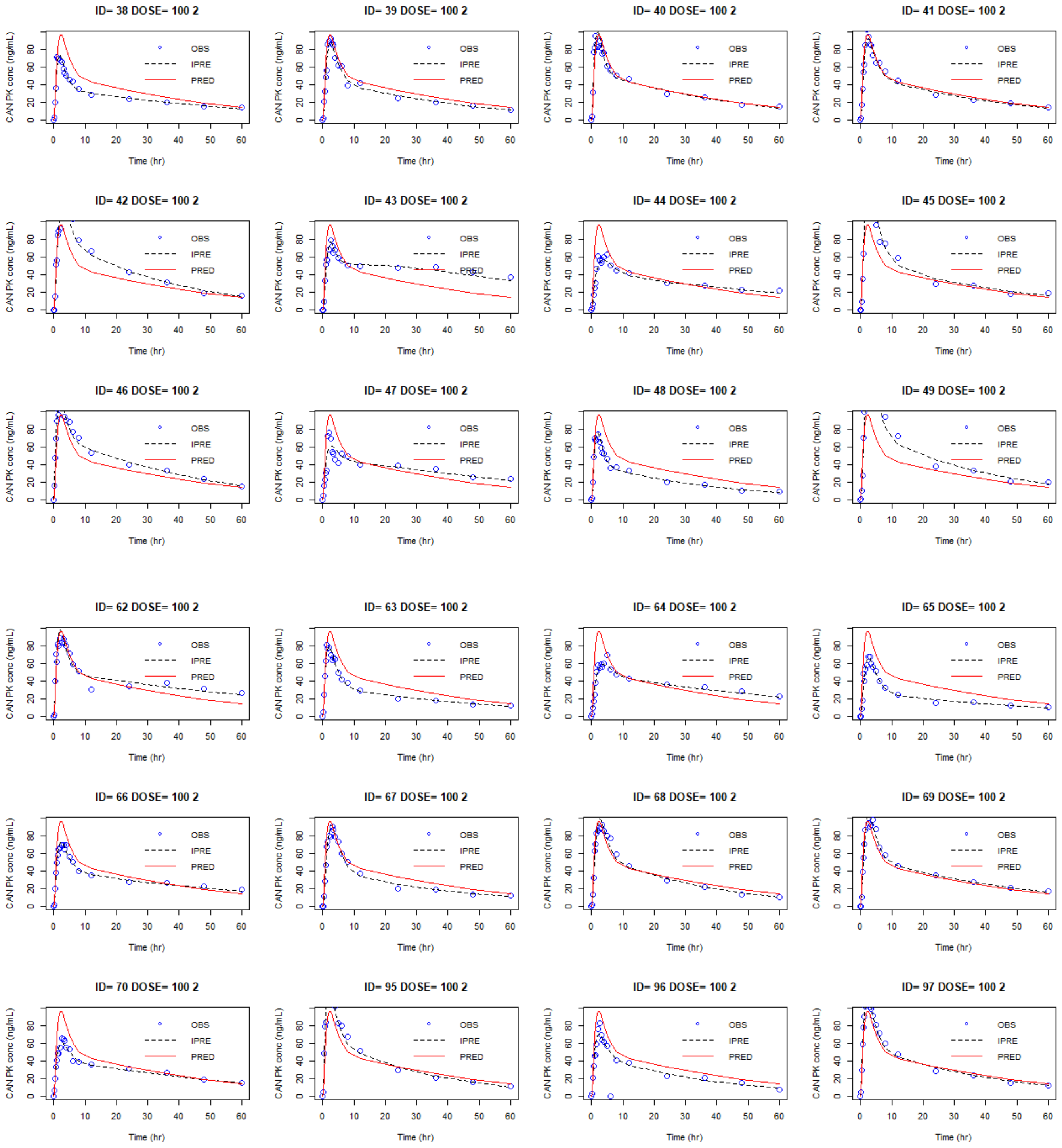
ID= 38 DOSE= 100 2
ID= 39 DOSE= 100 2
ID= 40 DOSE= 100 2
ID= 41 DOSE= 100 2
ID= 42 DOSE= 100 2
ID= 43 DOSE= 100 2
ID= 44 DOSE= 100 2
ID= 45 DOSE= 100 2
ID= 46 DOSE= 100 2
ID= 47 DOSE= 100 2
ID= 48 DOSE= 100 2
ID= 49 DOSE= 100 2
ID= 62 DOSE= 100 2
ID= 63 DOSE= 100 2
ID= 64 DOSE= 100 2
ID= 65 DOSE= 100 2
ID= 66 DOSE= 100 2
ID= 67 DOSE= 100 2
ID= 68 DOSE= 100 2
ID= 69 DOSE= 100 2
ID= 70 DOSE= 100 2
ID= 95 DOSE= 100 2
ID= 96 DOSE= 100 2
ID= 97 DOSE= 100 2
OBS
IPRE
PRED
CAN PK conc (ng/mL)
Time (hr)

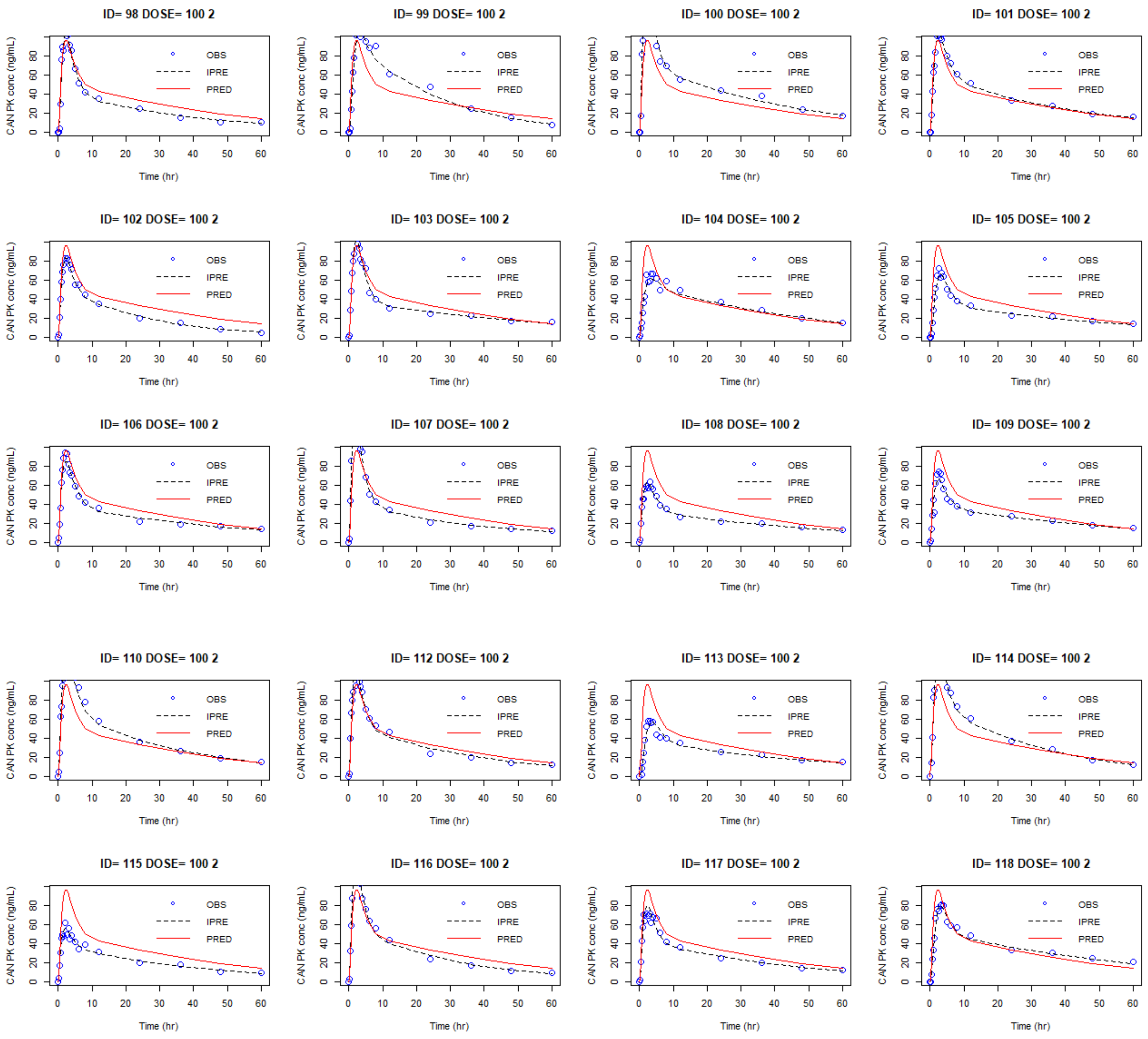

Figure S8. Observed (DV), individual predicted (IPRED) and population predicted (PRED) concentrations versus time estimated from the final model of **CAN.**

Comment on Figure S8: No apparent trend was found for the relationship between the individual residuals of the ETAs calculated by the base parent-metabolite model and covariates tested. The model was therefore adopted for further analysis.

(a) Demographic covariates

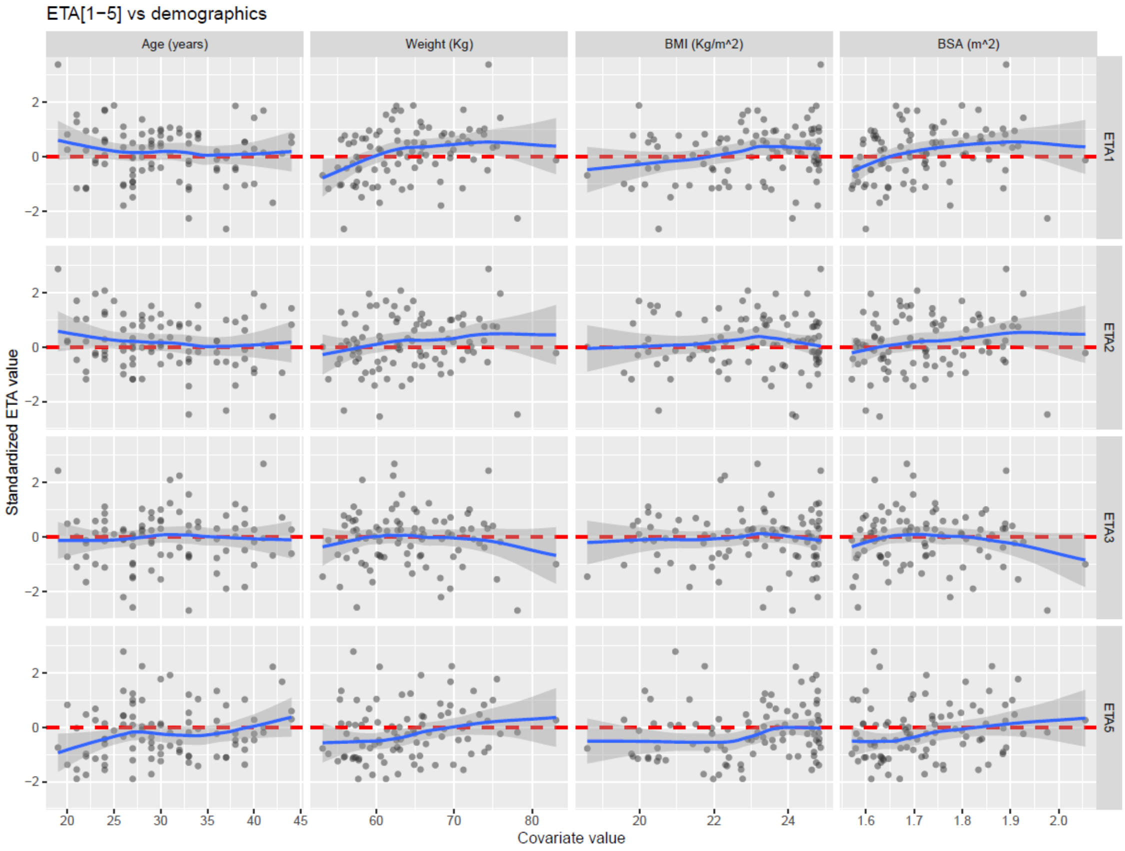

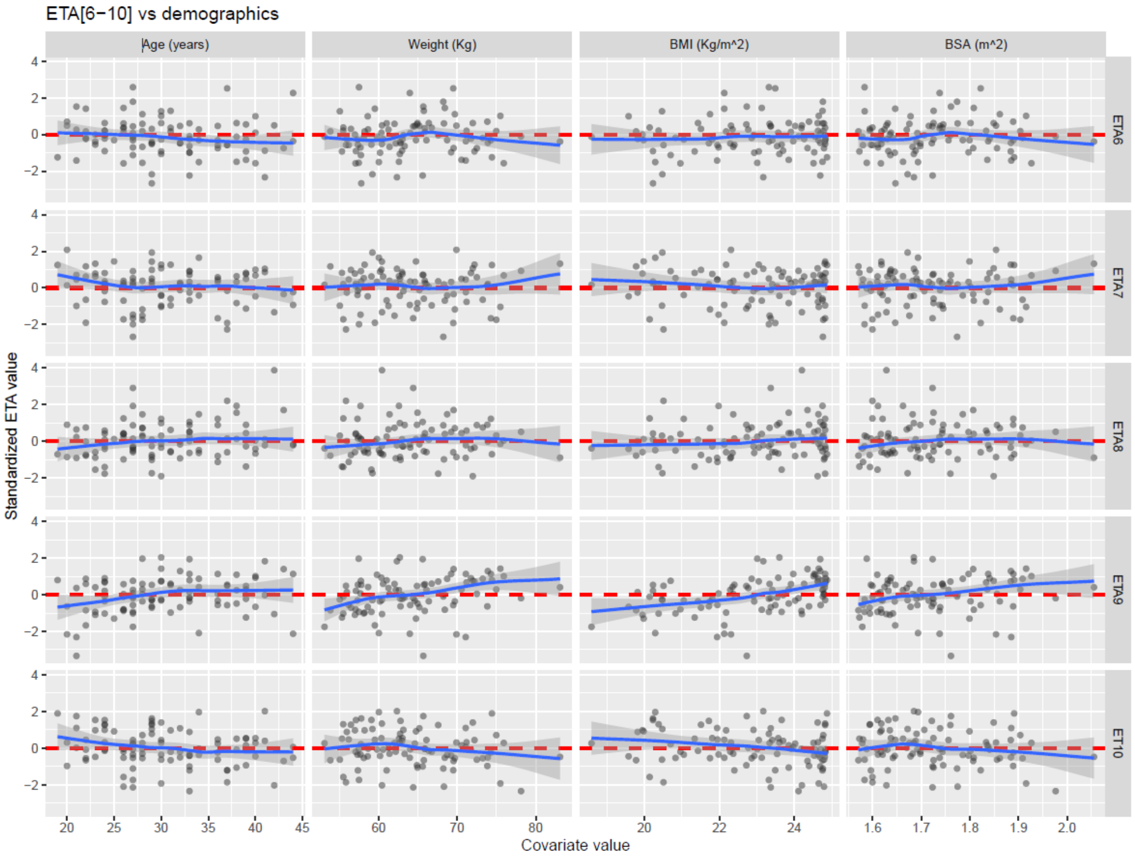

(b). Clinical covariates

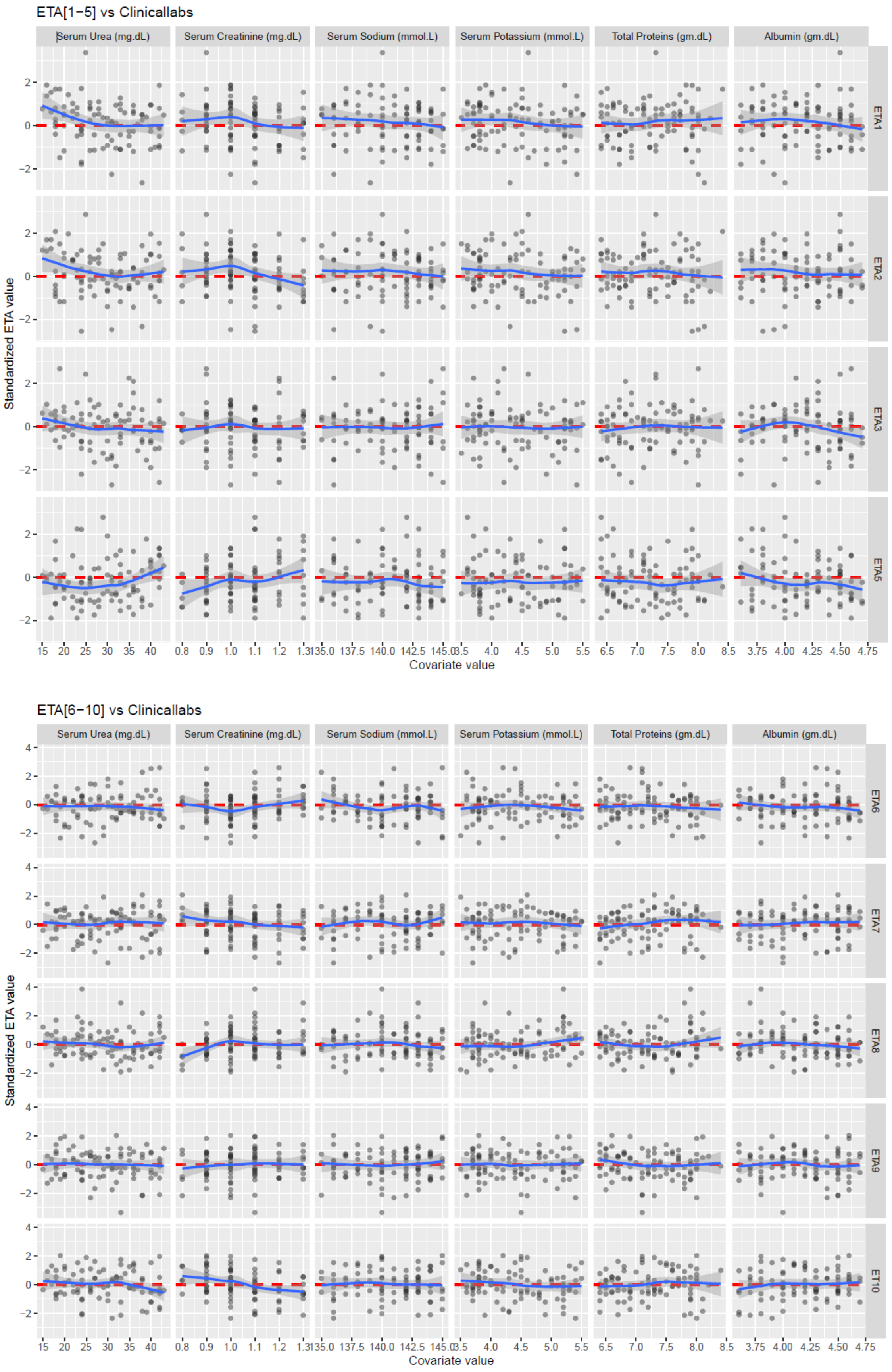

Figure S9. Relationship between ETAs and covariates (a) demographics and (b) clinical covariates.

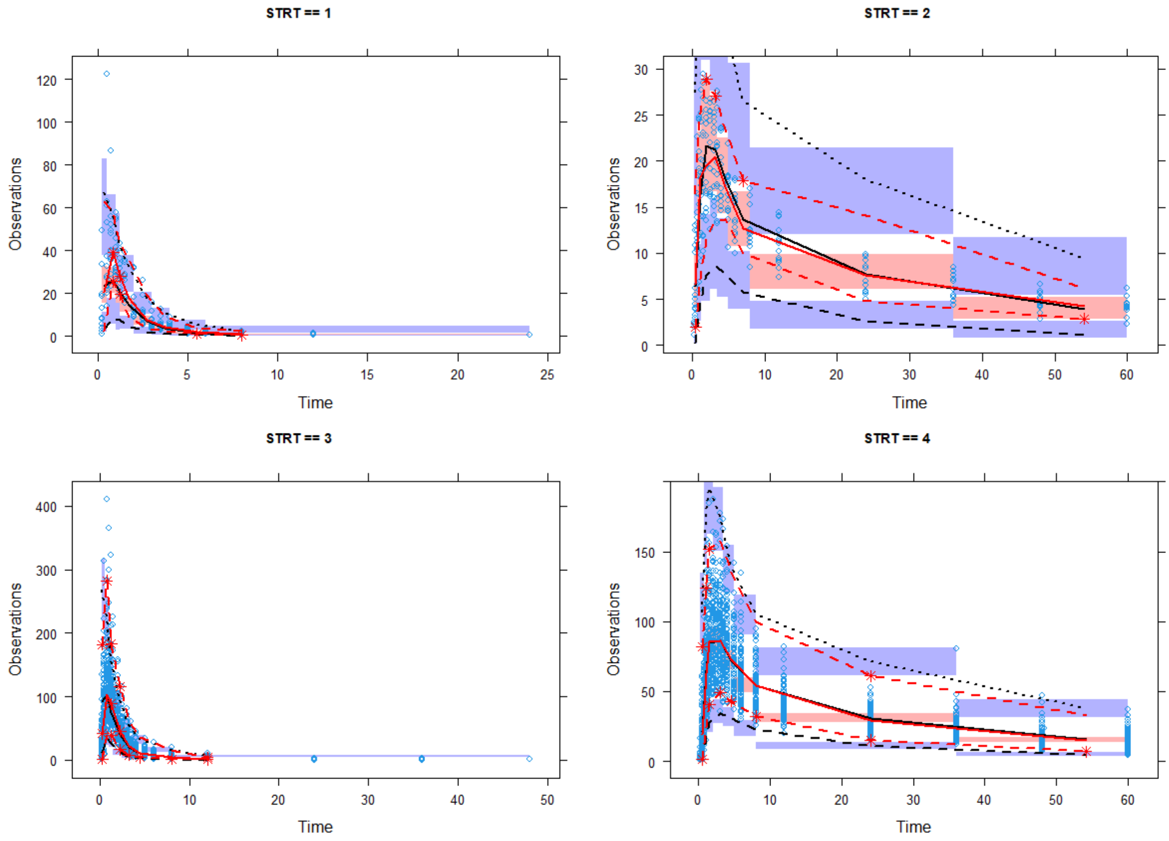

Figure S10. Visual predictive check for SPIR (left panel) and CAN (right panel) after the 25 mg dose (top panel) and 100 mg dose (bottom panel). STRT = 1 and 3 reflects SPIR profile with 25 mg and 100 mg, respectively; STRT = 2 and 4 reflects CAN profile with 25 mg and 100 mg, respectively. The red and black solid and dashed lines represent the median and upper and lower 95th percentile of the observations and model predictions, respectively. The data are overlaid as open blue circles. The simulated data from the model are shown as solid pink and blue regions which are the 95th confidence intervals around the median (red solid line) and 95th percentile interval of the model predicted data, respectively. The discrepancy between model simulations and observations pointed out with red (*).

The final model qualification using VPC is shown in Figure S7 for the 25 and 100 mg of the oral suspension (top and bottom panels, respectively) and for SPIR and CAN (left and right panels, respectively). In general, the model was able to adequately predict the observations for both doses and analytes, although there is a little over-prediction of the between-subject variability for CAN at the 25 mg dose (upper right panel). However, the median behavior was adequately predicted and the model was considered appropriate for the purpose of the study. Plot was truncated to 24 h to facilitate the visualization.

Section 2: Additional Information on the Simulation Based Exploration of what–if Scenarios in Fasted and Fed Cirrhotic and Non-Cirrhotic Subjects

2.1. Sensitivity Analysis to Find Main Changed in PK Parameters

2.1.1. Effect of Cirrhosis

In order to understand which PK parameters and what extent they change under the influence of cirrhosis, published studies using by Gardiner et al. [5] and Sungailia et al. [6] were referred. As per Gardiner et al., administration of 100 mg dose of Aldactone tablets in healthy volunteers has resulted in half-lives ($t_{1/2}$) 1.4 and 16.5 hours for SPIR and CAN, respectively, whereas Sungaila et al., reported that the same doses in cirrhotic subjects yielded 9.04 and 57.8 hours of half-life. The reason why Gardiner was used for comparison, instead of current available data on healthy adults from the clinical trials, is because accounting for the year of publication and the used formulation, this comparison would reflect more reliably the magnitude of changes in the PK between both populations. For the implementation of the cirrhotic scenario, it was assumed that the reduced half-life is governed solely by a reduction in clearance values for

both SPIR and CAN. This assumptions is reasonable as it is known that cirrhosis induced changes in the functional liver tissue results in drop in the drug kinetics related to enzymatic as well as the protein binding aspects [7].

Under this setting the corresponding % of drop in the clearances were 84.55% for SPIR and 71.55 for CAN. As in case of SPIR, its elimination is governed by complex metabolic pathways, the CL (elimination of the central compartment) is a combination metabolic pathways other than that forms CAN. Whereas in the case of CAN, the reduced clearance is served by its elimination clearance from the central compartment (CL_{CAN-0}). While performing sensitivity analysis by implementing these changes in the parameter values (i.e., −84.55% in CL and $L_{SPIR-CAN}$, −71.55% in CL_{CAN-0}) the resulted C_{max} values especially for CAN was unreasonably high (i.e., from ~ 3 to 45 hours). Based on Sungaila et al. it was observed that there would be a minimal or no change in the C_{max} values as compared to that in healthy when administered with 100 mg of Aldactone. Therefore the reduction in $CL_{SPIR-CAN}$ was optimized in such a way that there is no change in the C_{max} values as compared to that model predictions in healthy/non-cirrhotic scenario. Table S1 shows the finalized parameter values along with literature values of $t_{1/2}$ in healthy and cirrhotic conditions.

2.1.2. Effect of Food

Food effect on SPIR kinetics was evaluated based on the food effect study (i.e., Study 084-15, see Table 1 in the main text). As per this cross over study, it was identified that food reduced C_{max} (22.5% for SPIR and 25% for CAN) and increased the T_{max} (168% for SPIR and 113% for CAN) and AUCs (84% for SPIR and 13% for CAN). These observations are tabulated in Table S3. It is well known that SPIR is a BCS class-2 drug with poor aqueous solubility [8] and food has positive effect on its bioavailability [9]. This information and study data gives a strong impression that food behaves like a depot compartment to delay gastric emptying, and allows a slow and sustained release of the drug. This should likely to drop the rate of absorption, hence the C_{max}, and increase the T_{max} values. Under this setting it is also likely that the metabolic pathway to CAN would saturate which could alter the extent of the conversion from SPIR to CAN.

The sensitivity analysis provided insights that the slow absorption rate, which drops below the elimination rate, limits the elimination SPIR therefore follows flip-flop kinetics. This assumption driven changes, specifically a drop in absorption rate constant (ka) by 94.5%, 2 fold increase in bioavailability fraction (using a factor f) and ka mediated elimination adequately addressed the reduced C_{max} and increased T_{max} and AUCs. A drop in the fraction or extent of SPIR metabolism to CAN (Fm) from 0.7 to 0.42 has provided the observed changes in CAN kinetics. This information is tabulated under Table S4. It is important to make note that, due to unavailability of intra venous PK data SPIR its bioavailability (F) is structurally unidentifiable. Therefore, F was indirectly estimated in the form of apparent parameter (for instance, CL/F). So, food effect mediated 2 fold change in the F is governed by a factor (here denoted as f).

2.2. Simulation Based Evaluation of Different Doses in Cirrhotic and Non-Cirrhotic Pediatric Age Groups with/out Food Effect

For the purposes of dose selection and exploration of clinical scenarios (i.e., cirrhosis and food effect) 4 scenarios (including the one for dose selection) were simulated: (1) Non-cirrhotic in fasting state, (2) Cirrhotic in fasting state, (3) Non-cirrhotic in fed state and (4) Cirrhotic in fed state.

For these scenarios, the rate constants were carefully evaluated in adult subject to make sure each compartment maintain appropriate input-output relationship (see Table S4). Then for each set of scenarios the magnitude of changes in the parameter values that attributed to altered PK of SPIR and CAN in clinical scenarios (i.e., cirrhosis and food effect) in adults were assumed to be the same in pediatric subjects. It was also assumed that, between subject variability in these age groups will be similar to that in adults. Under these assumptions the corresponding final parameter estimates from the adults (see Table 3) were allometrically scaled to the median (50th percentile) of the body weights of 2, 6, 12 and 17 years old male and female (pediatric) subjects. The median body weights were collected from CDC growth charts (https://www.cdc.gov/growthcharts/html_charts/wtageinf.htm). Using the scaled population parameters in pediatric subjects, 200 virtual subjects of parameter values (100 for male and 100 for female) were generated using multivariate normal random distribution, then the model was implemented in MATLAB (MathWorks®) for the concentrations and post-processing to compute secondary PK parameters (i.e., AUC etc.).

Table S1. Literature-informed changes in spironolactone and canrenone clearance to reproduce the changes in the pharmacokinetic metrics in adult patients with liver cirrhosis.

Population		SPIR		CAN		Implementation in the model based on sensitivity analysis	SPIR		CAN
		$t_{1/2}$ (h)	% change in k_{SPIR-0}	$t_{1/2}$ (h)	% change in k_{CAN-0}		CL	$CL_{SPIR-CAN}$	CL_{CAN-0}
Healthy Subjects	Literature Data	1.4	0	16.5	0		629	217	17
Cirrhotic Subjects		9.04	−85.55%	57.8	−71.55%		97.9	135	4.8

$t_{1/2}$, plasma half-life; k_{SPIR-0}, elimination rate constant for SPIR central compartment, k_{CAN-0}, elimination rate constant for CAN central compartment; CL, SPIR elimination clearance from central compartment; $CL_{SPIR-CAN}$, metabolic clearance of SPIR to CAN; CL_{CAN-0}, CAN elimination clearance from central compartment.

Table S2. Comparison between various kinetic parameters in fasting and fed conditions in study 084-15.

Fasted/Fed	SPIR						CAN					
	T_{max}	% relative ratio	C_{max}	% relative ratio	AUC	% relative ratio	T_{max}	% relative ratio	C_{max}	% relative ratio	AUC	% relative ratio
Fasted	0.89	100	129.2	100	273.5	100	2.3	100	100.7	100	2504	100
Fed	2.39	268	100.1	77.5	505.6	184	4.9	213	75.64	75.1	2834	113

Table S3. Sensitivity analysis based parameter values that optimized to produce the changes with food effect on SPIR PK in adults.

Parameters	SPIR		CAN	
	**f**	**ka**	**k**	**Fm**
Values in fasting state	1	5.22	1.22	0.7
Values changed to in fed state	2	0.34 (0.065 × 5.22)	0.34 (since k >> ka)	0.42 (0.6 × 0.7)

F, factor for the change in the bioavailability of SPIR; ka absorption rate constant for SPIR; k, SPIR elimination rate constant; Fm, fraction metabolized from SPIR to CAN.

Table S4. Rate constants applied for SPIR and CAN kinetics in fasting and fed conditions in cirrhotic and non-cirrhotic groups.

Parameter	Fasting in non-cirrhotic	Fed in non-cirrhotic	Fasting in cirrhotic	Fed in cirrhotic
ka	5.22	0.34(0.065 × 5.22)	5.22	0.34(0.065 × 5.22)
k_{SPIR-0}	1.22 ($ka \gg k_{SPIR-0}$)	0.34 ($ka \ll k_{SPIR-0}$→ $k_{SPIR-0} = ka$)	0.19 (0.156x1.22)	0.19 ($ka > k_{SPIR-0}$)
$k_{SPIR-CAN}$	0.42 ($ka \gg k_{SPIR-CAN}$)	0.42*	0.27 (0.62x0.42) ($ka \gg k_{SPIR-CAN}$)	0.27 ($ka \gg k_{SPIR-CAN}$)
k_{CAN-0}	0.09 ($ka \gg k_{CAN-0}$)	0.09 ($ka \gg k_{CAN-0}$)	0.02 (0.28x0.09) ($ka \gg k_{CAN-0}$)	0.02 ($ka \gg k_{CAN-0}$)

ka, absorption rate constant; k_{SPIR-0}, elimination rate constant for SPIR from central compartment, $k_{SPIR-CAN}$, metabolic rate constant for the conversion from SPIR to CAN; k_{CAN-0}, elimination rate constant for CAN from central compartment; * This value for $k_{SPIR-CAN}$, might appear $ka < k_{SPIR-CAN}$ but still the input-out relationship is appropriate. To be specific, after applying f (factor for the change in the bioavailability of SPIR, in this case 2) and Fm (fraction metabolized from SPIR to CAN, in this case 0.7) input (i.e., 2 × 0.34 = 0.68) > output (i.e., 0.7 × 0.42 = 0.294).

Section 3: Evaluation of the Performance of the Proposed d-Optimally Designed Pediatric Study

The goal of this analysis was to evaluate if the suggested sparse sampling schedule can support estimation of the model parameters, and make sure that the values of the disposition parameters are comparable with that of the original model.

In this analysis the proposed study design (see section 3.5 in the main manuscript for details) was evaluated by simulation and estimation (SIM-EST) approach using NONMEM (double precision, version 7.3 ICON Development Solutions, Ellicott City, Maryland). The SIM-EST approach was implemented in two steps: (1) simulation of pediatric data from the final POPPK model and proposed study design, then (2) estimation of the parameters using the model and the simulated dataset. In the first step, a dataset was simulated using the sampling schedule and number of subjects specified in Table 6 (in the main manuscript) for the 0.5 mg/kg and 1.5 mg/kg doses in the subgroups 1.1, 1.2, 2.1, 2.2, 3.1 and 3.2. These simulations were performed with $SIM option. Overall the simulated data set included 36 subjects with 192 post dose samples within the proposed sampling windows. In the second step, the final POPPK model was used to estimate the parameters for the simulated dataset.

The SIM-EST analysis results showed that all the parameter values, from the sparse dataset, are very similar to those from the original model with % bias lower than 20% except for the absorption rate constant (showed in the Table S6). Moreover, all the parameters were estimated with reasonable precision (RSE <35% for the structural parameters and RSE<50 for the random parameters). The higher bias on the absorption rate constant was somehow expected, considering the large variability in this PK parameter and the sparse sampling strategy. However, to evaluate the clinical impact of a possible miss-estimation of the Ka a simulation was then performed using both models for the 0.5 and 1.5 mg/kg doses. The purpose of this comparison was to evaluate whether the output of both models lead to different PK metrics and different conclusion on the drug exposure in pediatrics. The results are shown in Figure S-11 for the extreme ages, i.e., adults and 2 years old children as a bracketing approach for the intermediate ages.

Table S5. Comparison of the parameter estimates from the original and sparse sampling model.

Parameter	Original Model		Sparse Sampling Model		% Bias
	Value	% RSE	Value	% RSE	
ALAG1	0.156	0.7	0.148	17.8	−5.1
ka (1/h)	5.22	1.1	8.37	34.3	60.3
CL (L/h)	629	3.3	674	23.3	7.2
V2 (L)	517	2.0	599	11.2	15.9
Q (L/h)	89.9	2.2	88.2	20.5	−1.9
V3 (L)	777	1.9	723	13.8	−6.9
Fm	0.7 FIX	-	0.7 FIX	-	
CLM1 (L/h)	217	4.1	234	28.3	7.8
CLM (L/h)	17	3.6	17.9	26.1	5.3
V4 (L)	189	3.4	226	22.9	19.6
Q1 (L/h)	60	3.9	59.3	30	−1.2
V5 (L)	448	3.0	537	27.6	19.9
**Between Subject Variability**					
ka (1/h)	97%	13.1	120.4%	19.1	24.1
CL (L/h)	40.7%	9.9	41%	48.5	0.7
V2 (L)	34.4%	11.6	21.5%	72.1	−37.5
CL, V2 (covariance)	80%	0.02	96.7%	0.05	20.8
Q (L/h)	28.4%	14.5	27.2%	31.3	−4.2
CLM1 (L/h)	42.8%	13.3	27.6%	27	−35.5
CLM (L/h)	28.1%	13	34.2%	16.8	21.7
V4 (L)	17.4%	22.4	17.6%	102.9	1.1
Q1 (L/h)	26.3%	17.5	30.1%	43.5	14.4
V5 (L)	29.8%	11.6	36.9%	21.7	23.8
**Residual Error**					
EPS1	29.1%	2.8	32.6%	13.3	12
EPS2	13.3%	1.3	13.1%	17	−1.5

CL, apparent clearance of SPIR from central compartment; CLM1, apparent metabolic clearance of SPIR to CAN; Fm, fraction metabolized from SPIR to CAN; h, hour; L, litre; ALAG1, lag time; ka, absorption rate constant; Q, apparent intercompartmental clearance of SPIR; RSE, relative standard error; V2, apparent central volume of distribution of SPIR; V3, peripheral volume of distribution of SPIR; CLM, apparent clearance of CAN from central compartment; V4, apparent central volume of distribution of CAN; V5, apparent peripheral volume of distribution of CAN; Q1, apparent intercompartmental clearance of CAN; EPS1, proportional residual error for SPIR; EPS2, proportional residual error for CAN. %Bias = 100/Parameter_original × (Parameter_sparse-sampling − P_original)

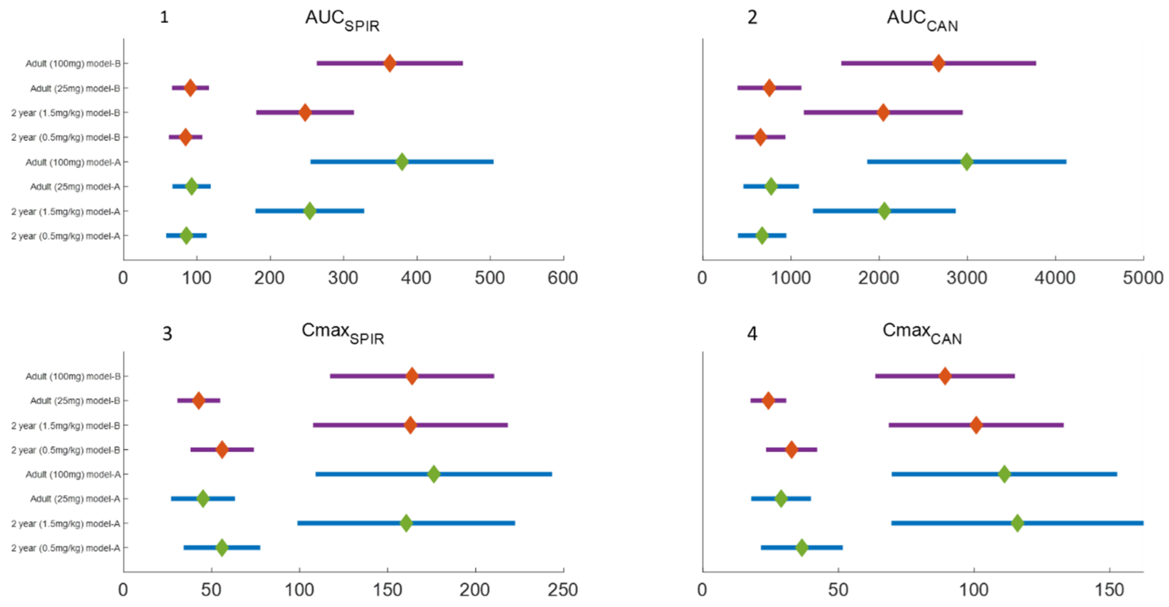

Figure S11. Comparison of the PK metrics from model A (original model) and B (sparse sampling model). Panel 1: AUC of SPIR; 2: AUC of CAN; 3: Cmax of SPIR; 4: Cmax of CAN. The green diamonds and solid blue lines reflect the mean and (±) SD of the different PK metrics in adults and 2 years old children after different doses with model A. The orange diamonds and solid purple lines reflect the mean and (±) SD of the different PK metrics in adults and 2 years old children after different doses with model B.

Both models led to very similar PK metrics in the different age-based populations and the C_{max} of CAN was the most sensitive metric to differences in the PK estimates. Based on this analysis, the study design was considered appropriate to characterize the PK of SPIR and CAN in pediatrics as possible miss-estimation of the K_a using this design will not lead to different conclusion regarding PK outcomes.

References

1. Food and Drug Administration. FDA Guidance for Industry, Population Pharmacokinetics. 2019; CP1. https://www.fda.gov/media/128793/download. (accessed on 27 February 2021).
2. Byon, W.; Smith, M.K.; Chan, P.; Tortorici, M.A.; Riley, S.; Dai, H.; Dong, J.; Ruiz-Garcia, A.; Sweeney, K.; Cronenberger, C. Establishing best practices and guidance in population modeling: an experience with an internal population pharmacokinetic analysis guidance. *CPT Pharmacometrics Syst. Pharmacol*. **2013**, *2*, e51.
3. Ette, E.I.; Williams, P.J.; Kim, Y.H.; Lane, J.R.; Liu, M.J.; Capparelli, E.V. Model appropriateness and population pharmacokinetic modeling. *J. Clin. Pharmacol.* **2003**, *43*, 610–623.
4. Ette, E.I.; Williams, P.J.; Lane, J.R. Population pharmacokinetics III: design, analysis, and application of population pharmacokinetic Studies. *Ann. Pharmacother.* **2004**, *38*, 2136–2144.
5. Gardiner, P.; Schrode, K.; Quinlan, D.; Martin, B.K.; Boreham, D.R.; Rogers, M.S.; Stubbs, K.; Smith, M.; Karim, A. Spironolactone metabolism: steady-state serum levels of the sulfur-containing metabolites. *J. Clin. Pharmacol.* **1989**, *29*, 342–347.
6. Sungaila, I.; Bartle, W.R.; Walker, S.E.; DeAngelis, C.; Uetrecht, J.; Pappas, C.; Vidins, E. Spironolactone pharmacokinetics and pharmacodynamics in patients with cirrhotic ascites. *Gastroenterology* **1992**, *102*, 1680–1685.
7. Rodighiero, V. Effects of liver disease on pharmacokinetics. An update. *Clin. Pharmacokinet.* **1999**, *37*, 399–431.
8. Custodio, J.M.; Wu, C.Y.; Benet, L.Z. Predicting drug disposition, absorption/elimination/transporter interplay and the role of food on drug absorption. *Adv. Drug Deliv. Rev.*, **2008**, *60*, 717–733.
9. O'Shea, J.P.; Holm, R.; O'Driscoll, C.M.; Griffin, B.T. Food for thought: formulating away the food effect - a PEARRL review. *J. Pharm. Pharmacol.*, **2019**, *71*, 510–535.
